# Predictive Modelling of Current and Future Potential Distribution of the Spectacled Bear (*Tremarctos ornatus*) in Amazonas, Northeast Peru

**DOI:** 10.3390/ani10101816

**Published:** 2020-10-06

**Authors:** Gerson Meza Mori, Elgar Barboza Castillo, Cristóbal Torres Guzmán, Dany A. Cotrina Sánchez, Betty K. Guzman Valqui, Manuel Oliva, Subhajit Bandopadhyay, Rolando Salas López, Nilton B. Rojas Briceño

**Affiliations:** 1 Instituto de Investigación para el Desarrollo Sustentable de Ceja de Selva (INDES-CES), Universidad Nacional Toribio Rodríguez de Mendoza de Amazonas (UNTRM), Chachapoyas 01001, Peru; ebarboza@indes-ces.edu.pe (E.B.C.); cristobal.torres@untrm.edu.pe (C.T.G.); alexander.cotrina@untrm.edu.pe (D.A.C.S.); betty.guzman@untrm.edu.pe (B.K.G.V.); soliva@indes-ces.edu.pe (M.O.); rsalas@indes-ces.edu.pe (R.S.L.); 2 Department of Ecology and Environmental Protection, Poznan University of Life Sciences, Piatkowska 94, 60-649 Poznan, Poland; subhajit.bandopadhyay@up.poznan.pl

**Keywords:** andean bear, biogeography, conservation, deforestation, ecological niche model (ENM), MaxEnt, protected areas, species distribution model (SDM)

## Abstract

**Simple Summary:**

The spectacled, or Andean, bear (*Tremarctos ornatus*) is threatened by human activities, despite being a key species for conservation. In our study, the sightings and tracks for spectacled bear were collected, key environmental variables affecting its distribution were identified, and its distribution was predicted under both current and future (2050 and 2070) conditions in Amazonas, northeastern Peru. Under current conditions, areas with “high”, “moderate” and “low” probability for the spectacled bear distribution cover about 1.99% (836.22 km^2^), 14.46% (6081.88 km^2^) and 20.73% (8718.98 km^2^) of Amazonas, respectively. Under all future conditions, the “high” probability area will increase, while the “moderate” and “low” probability areas, as well as total area (sum of “high”, “moderate” and “low”), will decrease. The, protected natural areas in Amazonas, currently and in the future, do not cover most of the important habitats for the spectacled bear. Therefore, to effectively conserve this species, it is strongly recommended that areas with “high” (even “moderate”) probability and the main ecosystems it inhabits should be designated as priority areas for research and conservation (even in natural protected areas). We assume that our study will make a strong contribution towards the sustainable conservation for spectacled bear under such threaten conditions.

**Abstract:**

The spectacled, or Andean, bear (*Tremarctos ornatus*) is classified as vulnerable by the IUCN due to climate change and human-induced habitat fragmentation. There is an urgent need for the conservation of spectacled bear at real time. However, the lack of knowledge about the distribution of this species is considered as one of the major limitations for decision-making and sustainable conservation. In this study, 92 geo-referenced records of the spectacled bear, 12 environmental variables and the MaxEnt entropy modelling have been used for predictive modelling for the current and future (2050 and 2070) potential distribution of the spectacled bear in Amazonas, northeastern Peru. The areas of “high”, “moderate” and “low” potential habitat under current conditions cover 1.99% (836.22 km^2^), 14.46% (6081.88 km^2^) and 20.73% (8718.98 km^2^) of the Amazon, respectively. “High” potential habitat will increase under all climate change scenarios, while “moderate” and “low” potential habitat, as well as total habitat, will decrease over the time. The “moderate”, “low” and total potential habitat are distributed mainly in Yunga montane forest, combined grasslands/rangelands and secondary vegetation and Yunga altimontane (rain) forest, while “high” potential habitat is also concentrated in the Jalca. The overall outcome showed that the most of the important habitats of the spectacled bear are not part of the protected natural areas of Amazonas, under current as well as under future scenarios.

## 1. Introduction

The spectacled, or Andean, bear (*Tremarctos ornatus*) is the only South American representative from the family *Ursidae* [1]. The spectacled bears are living within a wide range of altitudes (~200–4750 m a.s.l.) and their habitats mostly found at the tropical rainforest, dry forest, montane forest, cloud forest, herbaceous paramo and in others regimes along the three Andean mountain ranges covering Venezuela to Colombia, Ecuador, Peru, Bolivia and Argentina [2,3,4]. Considering its widespread distribution, we found that, spectacled bear is the key habitat species for conservation [5,6] and an ambassador for the umbrella species [2,7]. However, human colonization and poaching along with the road expansion, agricultural and forest activities are responsible for the sharp decline of the species’ population. This situation accelerates the human-bear conflicts, poaching and illegal trade, which leads the fragmentation of the species’ habitat conservation areas [8,9,10]. Thus it is currently listed a global ”vulnerable” habitat species by IUCN [11,12] and in Peru [10,13], and in the CITES Appendix I of Peru, because its trade is subject to particularly strict regulation so as not to endanger its survival and is authorized only in exceptional circumstances [14]. In general, the spectacled bear are the habitats in 30 Natural Protected Areas (NPA) in Peru [10,15]. However, insufficient information and knowledge gap on the distribution of the spectacled bear may be jeopardizing either the survival of local populations [16] or actual knowledge of the legally protected area under NPA systems (effectiveness of NPA).

The lack of a detailed distribution of this threatened species, little-known or invasive, even in historical or future scenarios, is a serious concern for the management and conservation of wildlife authority, considering this is one of the priorities for decision-making and conservation action plans [17,18]. Thus, Species Distribution Models (SDM), based on observed data on the presence of the species, predictive environmental variables, and statistical and cartographic procedures, are one of the most widely used tools for improving this knowledge [19]. In addition, SDMs have been increasingly applied in a variety of wildlife studies, and in ecology and conservation biology [18,20]. Among several SDMs, the most accurate maximum entropy model (MaxEnt) is considered as outperform compared to other SDMs in predictive accuracy, with extremely small sample size tolerance [21,22]. Moreover, the use of MaxEnt software is particularly easy to use [23] and has been widely used in studying habitats of different bear species that can be also used by wildlife officials [17,18,24,25,26,27,28,29].

In this study, we modelled both the current and future potential distribution of the spectacled bear in Amazonas region of northeastern Peru aiming to identify the possible changes in time-space consortium in the habitat of the species, as well as prioritizing areas for research and conservation. At first the base of georeferenced records of the species’ presence were constructed, second, the important and uncorrelated environmental variables were identified and selected, and finally the maps were modelled and the changes in the environmentally suitable area under current and future conditions for 2050 and 2070 were identified. These models were composed with the functional units of the Peruvian Ecosystem Maps, with the current IUCN spectacled bear distribution map and with Amazonas NPAs. Our study hypothesized that the highly suitable area for the spectacled bear is expected to decline in the future along with the changes in habitat functional units. This possibilities leads to IUCN map will omit the important habitat areas in the Amazonas, and that current NPAs are not being effective in legally protecting the species. Moreover, our study can be considered as an alert that will help to better understand spectacled bear distribution and habitat preferences in the region. This work can also serve as a tool for designing surveys looking at the location for new individuals and in future conservation and will help conservation management plans.

## 2. Materials and Methods

### 2.1. Study Area

**Figure 1:**
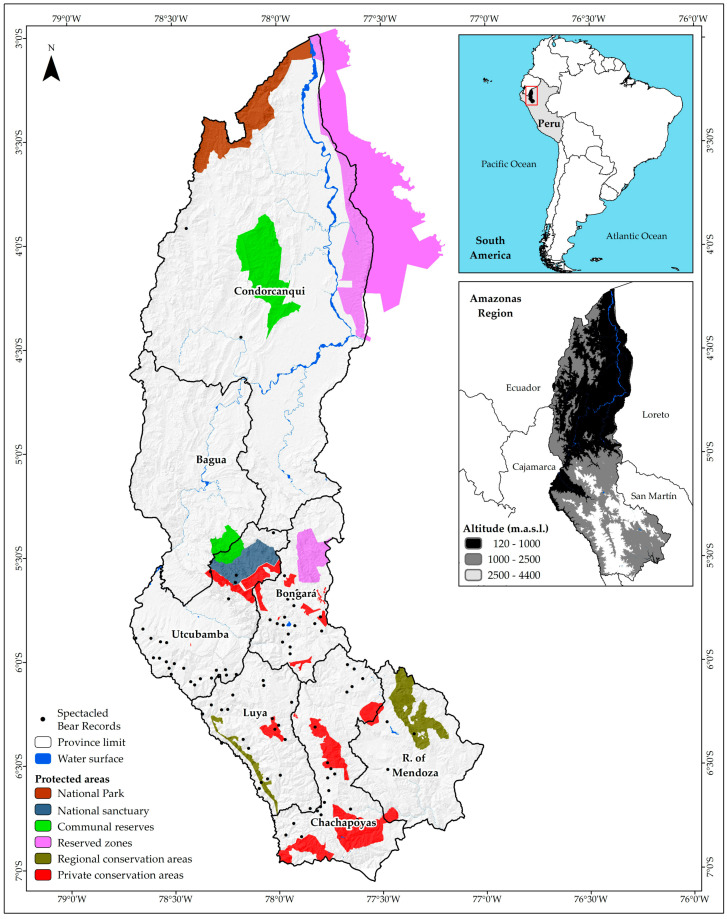
Geographical location and protected areas in Amazonia (Peru).

Amazonas region covers an estimated area of 42,050.37 km^2^ of rugged landscape, largely covered by the Amazon forest located in the northeastern Peru (Figure 1). The area covers the parallels 3°0′ and 7°2′ South latitude and the meridians 77°0′ and 78°42′ West longitude, with an altitudinal gradient of 120 m a.s.l. in the north and 4900 m a.s.l. in the south. This altitudinal gradient favors a high biophysical diversity. The National Map of Peruvian Ecosystems [30,31] has identified about 20 functional units, including anthropogenic: Bofedal (1.95 km^2^), Yunga Altimontane (Pluvial) Forest (3337.79 km^2^), Floodplain Alluvial Forest (681.62 km^2^), Yunga Basimontane Forest (16,455.08 km^2^), High Hill Forest (1717.38 km^2^), Low Hill Forest (3245.46 km^2^), Non-Floodplain Terrace Forest (268. 56 km^2^), Seasonally dry inter-Andean forest (936.38 km^2^), Yunga mountain forest (7988.75 km^2^), Island (30.74 km^2^), Jalca (1439.05 km^2^), Lake and lagoon (8.79 km^2^), Andean scrub (325. 42 km^2^), Palm Swamp (359.35 km^2^), Grasslands/Herbazales (1478.62 km^2^), River (408.51 km^2^), Varillal (18.26 km^2^), Secondary Vegetation (2936.23 km^2^), Agricultural Zone (392.59 km^2^) and Urban Zone (11.38 km^2^). This region characterized with ”hot and humid”, “dry hot” and “warm temperate and slightly humid”, with the maximum temperatures of 40 °C in the lowland forest to the north and minimum temperatures of 2 °C in the mountain ranges to the south. In some areas there is a water shortage of 924 mm/year, whereas in others there is a surplus of up to 3000 mm/year [32]. Politically, Amazonas is divided into seven provinces (Bagua, Bongará, Chachapoyas, Condorcanqui, Luya, Rodríguez de Mendoza and Utcubamba) and is the third region in the country with the greatest number of NPAs under six modalities [15]. There are one national park (884.77 km^2^), one national sanctuary (392.16 km^2^), two communal reserves (1185.65 km^2^), two reserved areas (4347.98 km^2^ shared with Loreto region), two regional conservation areas (628.74 km^2^) and 19 private conservation areas (1482.34 km^2^) (Figure 1).

### 2.2. Geo-Referenced Records of the Spectacled Bear

Georeferenced records of spectacled bear sightings and traces were used obtained from (i) participatory mapping and semi-structured interviews conducted with 38 villagers between 34 and 95 years old, in 19 communities in the Amazon; (ii) personal communications with local researchers; and (iii) virtual platform download from the Global Biodiversity Information Facility (GBIF) [33] through three QGIS Plugins version 10.3 (GBIF occurrences, Species Explorer and Natusfera). The MaxEnt models are based on the assumption that all locations (most importantly in the environmental space) are uniformly sampled [23]. In order to eliminate the spatial sampling bias and improve the model performance [34], we filtered the universe of georeferenced records to a 3 km grid [17]. The spatial filter reduced the georeferenced records from 163 to 92 (Figure 1).

### 2.3. Environmental Variables

Environmental variables were applied in this study based on the reported studies on potential distribution [17,28] and habitat [1,3] of the spectacled bear. Namely, 23 environmental variables were selected, including 19 bioclimatic, two topographic (altitude and slope), water availability and shelter availability. The shelter should be understood as areas within the forest where bears can find shade not only to avoid the midday heat, but also dens to give birth and shelter for nesting on the ground and in trees [28,35]. The bioclimatic layers with a spatial resolution of 30 s (~1 km) were obtained from the World Climate Geodatabase, WorldClim (http://worldclim.org). From this geodatabase, WorldClim version 2 [36] was used for bioclimatic information extraction under current conditions (average 1970–2000) and version 1.4 [37] was used for both 2050 (average 2041–2060) and 2070 (average 2061–2080) climatic information extraction for future time periods. For 2050 and 2070, four greenhouse gas emission scenarios based on the Representative Concentration Pathways (RCP) [38] of the Community Climate System Model see 4 (CCSM4) [39] were considered. Namely, RCP with a declining radiative level of 2.6 W/m^2^ (RCP 2.6), stabilized 4.5 W/m^2^ (RCP 4.5), intermediate 6.0 W/m^2^ (RCP 6.0) and increased 8.5 W/m^2^ (RCP 8.5) [40].

The topographic variables were derived from the 250-m spatial resolution Digital Elevation Model (DEM) downloaded from the CGIAR Consortium for Spatial Information portal (http://srtm.csi.cgiar.org/). This DEM has been generated from Shuttle Radar Topography Mission (SRTM) data [41]. Proximity to water availability was generated by the Euclidean distance algorithm with a spatial resolution of 250 m. The vector layer of the hydrography (rivers and lakes at a scale of 1:100,000) of the 6 h, 7g-h, 8f-h, 9f-h, 10f-h, 11f-h, 12f-h, 13f-i, 14g-i and 15 h quadrants of the National Chart of the National Geographic Institute (IGN, Madrid, Spain) was used. These were downloaded from the Ministry of Education website [42]. The cartographic gaps in these charts were filled with the hydrology of the Amazon Ecological and Economic Zone (ZEE-A) [43]. The percentage of forest cover per 100-m pixel of spatial resolution obtained from the Copernicus Global Land Service version 2 of 2015 was used for shelter availability [44].

Overall, 23 thematic layers were constructed for each evaluated scenario (one current and eight future) in ASCII format with 250-m spatial resolution. All non-bioclimatic variables were assumed to be unchanged for 2050 and 2070.

### 2.4. Selection of Environmental Variables

Collinearity between environmental variables may cause not only over-adjustment problems, but also increase uncertainty and decrease the statistical power of the model [45,46]. Owing to the importance of non-bioclimatic variables, the process of selection/elimination of variables by collinearity was only applied to bioclimatic variables [17]. Therefore, for the coordinates of the georeferenced records of the spectacled bear, pixel values were extracted from the 19 thematic layers of current bioclimatic variables [47]. Then, using the R programming language, (i) Pearson’s correlation coefficients (r) between the variables were calculated (Table S1), from which, (ii) the optimal number of clusters was determined using the Euclidean distances and the K-means clustering algorithm using factoextra package (Figure S1a) and (iii) the cluster dendrogram was then constructed (Figure S1b) [48]. This led to internally correlated groups of variables. For each group, the variable with the highest score in the jackknife test (Figure S3) was considered in a preliminary model generated using only the 19 bioclimatic variables. Finally, eight bioclimatic variables were selected: Mean Diurnal Range (bio02), Isothermality (bio03), Temperature Seasonality (bio04), Temperature Annual Range (bio07), Mean Temperature of Driest Quarter (bio09), Annual Precipitation (bio12), Precipitation of Driest Month (bio14) and Precipitation Seasonality (bio15).

### 2.5. Modeling Approach and Potential Distribution Changes

The potential distribution models were generated using the machine-learning algorithm applying the Maximum Entropy principle [49], implemented in the open source software MaxEnt version 3.4.1. The 75% and 25% of the georeferenced records (selected at random) were used for training and validation of each model, respectively [49]. The algorithm was run using 10 replicates in 1000 iterations with different random partitions (Bootstrap method), with a convergence threshold of 0.00001 and 10,000 maximum background points. Other settings were kept by default [50], since MaxEnt is able to select the appropriate function for the number of samples used for a model [23,45]. The models were validated using the Area Under the Curve (AUC) [49,51] method, calculated from the Receiver Operating Characteristic (ROC) [52]. Based on the AUC values, there were five differentiated levels of performance [53]: excellent (>0.9), good (0.8–0.9), accepted (0.7–0.8), bad (0.6–0.7) and invalid (<0.6). The main advantage of this method is the fact that a threshold is independent, and the assessment results are more objective [54]. The logistic output format was used for both current and future models [55]. This format generated a map of continuous probability values for the potential distribution ranging from 0 to 1. These were further reclassified into four ranges [56]: “high” (>0.6), “moderate” (0.4–0.6) and “low” (0.2–0.4), potential habitat, and ‘non-potential habitat’ (<0.2). In view of the conservation objectives of this work, low cut-off thresholds were considered in order to achieve a larger area and apply a precautionary principle.

Out of nine reclassified maps of spectacled bear potential distribution, including one under current conditions and eight under climate change scenarios for 2050 and 2070, it was possible to extract urban centers, roads and water bodies, to avoid the overestimate areas of suitability. By this point, urban centers were extracted from the ZEE-A [43], roads (national, regional and neighborhood road networks) were obtained from the Ministry of Transport and Communications [57] and water bodies were obtained from national maps [42] and the ZEE-A [43]. These maps were then superimposed on each other to determine changes in habitat ranges. The potential distribution map under current conditions was also compared with the current IUCN spectacled bear distribution map [11], which was developed by experts with knowledge of spectacled bear habitat needs and known occurrence records. In addition, using the IUCN map as a template, a set of nine maps were extracted and overlapped to determine changes in habitat ranges under these geographic boundaries.

### 2.6. Identification of Habitat Changes and Priority Areas for Research and Conservation

The nine maps were overlaid with the functional units of National Map of Ecosystems in Peru [30,31], obtained from the MINAM geoserver [58], to determine habitat (functional units) changes. In addition, were overlaid with the Peruvian NPA system [15] obtained from the SERNANP geoserver [59]. This was done to determine the effectiveness of these areas to legally protect the species and to select areas that may be prioritized for research and potential future conversion to protected areas.

## 3. Results

### 3.1. Model Performance and the Importance of Environmental Variables

A total of nine predictive models of the potential distribution of the spectacled bear have been obtained, including, one under current conditions and eight under climate change scenarios for 2050 and 2070. All models have AUC > 0.9 (Table 1), showing outstanding predictive performances. The best performing model was the 2070 RCP 6.0 model, with AUC = 0.915 ± 0.012.

**Figure 2:**
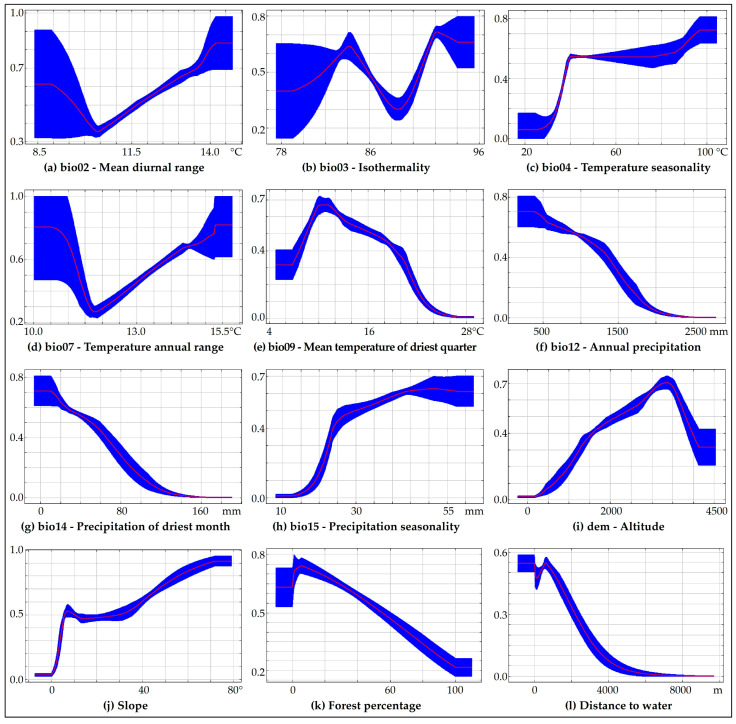
Mean response curves of the 10 replicated MaxEnt runs (red) and standard deviation (blue), showing the relationships between environmental variables and the probability of presence of the spectacled bear. If the °C of temperature (**a**–**e**), mm of precipitation (**f**–**h**), m a.s.l. (**i**), degrees of slope (**j**), percentage of forest (**k**) or distance to water (**l**) increase (*x*-axis), the probability of presence of the spectacled bear increases or decreases from 0 to 1 (*y*-axis).

The response curves (Figure 2) show the response of each environmental variable to the expected suitability, both in each variable and in the correlation with others. Under current conditions (Figure 2), results showed that the spectacled bear uses mostly sparsely wooded areas (Figure 2k) and prefers higher elevation zones (Figure 2i). The high slopes (Figure 2j) are not restrictive to spectacled bear distribution, though it does demand close proximity to water sources (Figure 2l).

In relation to bioclimatic variables, spectacled bear distribution decreases in areas with higher precipitation, in terms of annual precipitation (Figure 2f) or precipitation in the driest month (Figure 2g). It, furthermore, avoids areas with seasonal precipitation (Figure 2h) below 25 mm, in the same way with temperature (Figure 2c) less than 50 °C. The likelihood of the presence of the Andean bear has a similar response for the annual temperature range (bio7) and the mean day range (bio2). In other words, for both bioclimatic variables, from temperatures close to 10.5–12.0 °C the probability increases (in a straight line) as the temperature rises, until it reaches a maximum temperature of 14.0–15.5 °C (Figure 2a,d). The spectacled bear also prefers areas with near-peak isotherms of 78 °C and 92 °C (Figure 2b).

From 66.7% to 75.2% of the potential distribution of the spectacled bear, in all scenarios, was driven by three environmental variables: bio09 (mean temperature of driest quarter), bio14 (precipitation of driest month) and forest (percentage of forest cover) (Table 2). The environmental variables that were among the three of least contribution, in three or more models, were bio02 (mean diurnal range), bio03 (isothermality), bi04 (temperature seasonality), dem (elevation) and distance to water.

**Figure 3:**
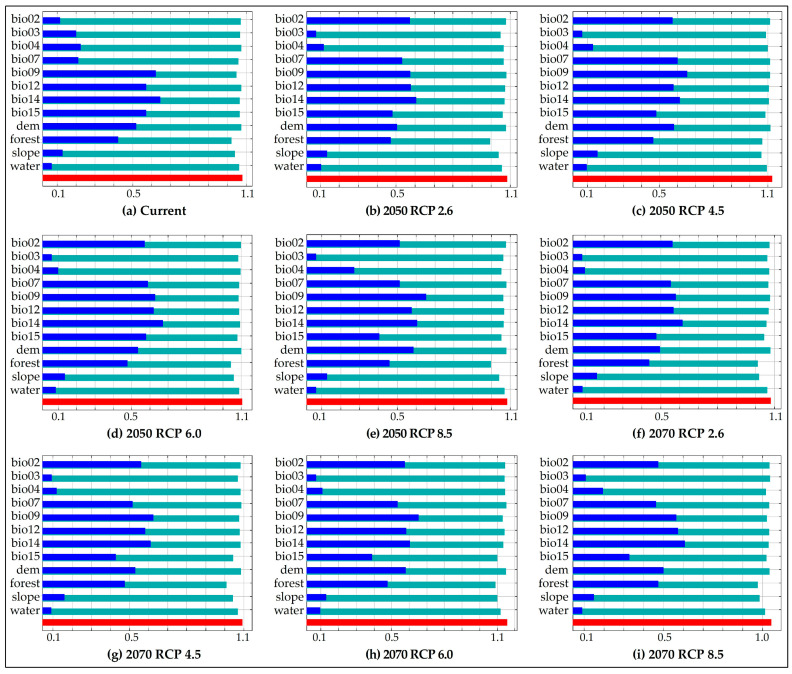
Jackknife test of regularized training gain of environmental variables to MaxEnt models under current conditions (**a**) and climate change scenarios in 2050 (**b**–**e**) and 2070 (**f**–**i**) of the spectacled bear in Amazonas (Peru). Regularized training gain without variable (green), with only variable (blue) and with all variables (red). Variables of greatest importance according to jackknife for the models: mean temperature of the driest room (bio09) and precipitation of the driest month (bio14).

The environmental variables that apparently have the most useful information by themselves are bioo14 (current, 2050 RCP 2.6 and 6.0, 2070 RCP 2.6 and 8.5) and bio19 (2050 RCP 4.5 and 8.5, 2070 RCP 4.5 and 6.0); whereas the environmental variables that seem to have the greatest amount of information lacking in the other variables are forest (current, 2050 RCP 2.6, 6.0 and 8.5, 2070 RCP 2.6–8.5) and slope (2050 RCP 4.5) (Figure 3).

### 3.2. Potential Current and Climate Change Scenario Distribution of the Spectacled Bear

**Figure 4:**
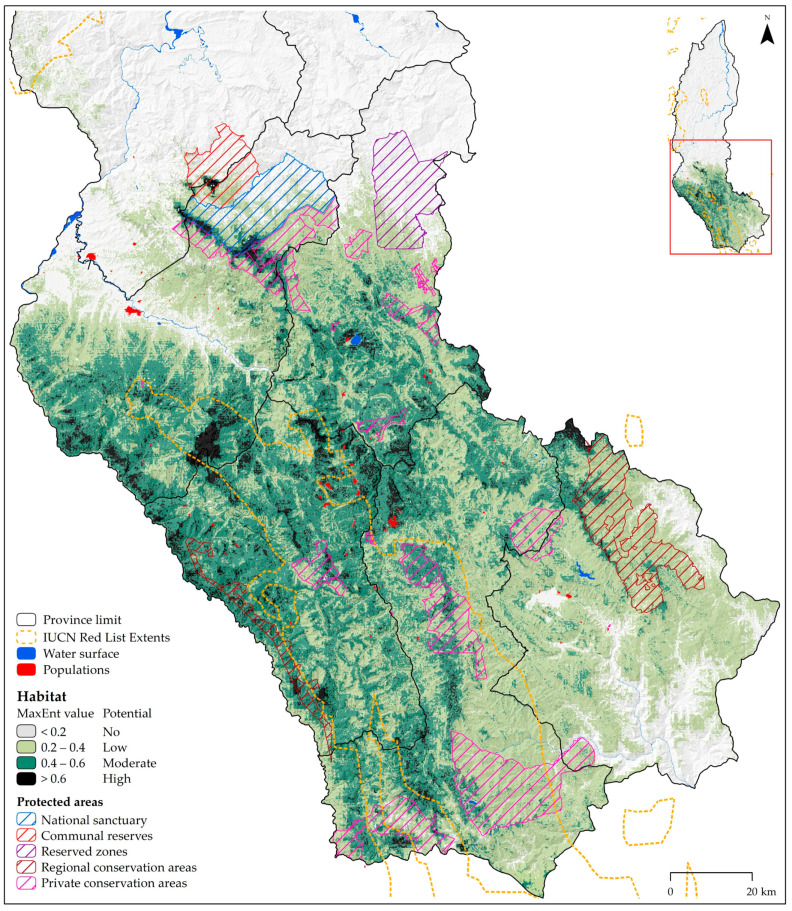
Current potential distribution of the spectacled bear in Amazonas (Peru).

**Figure 5:**
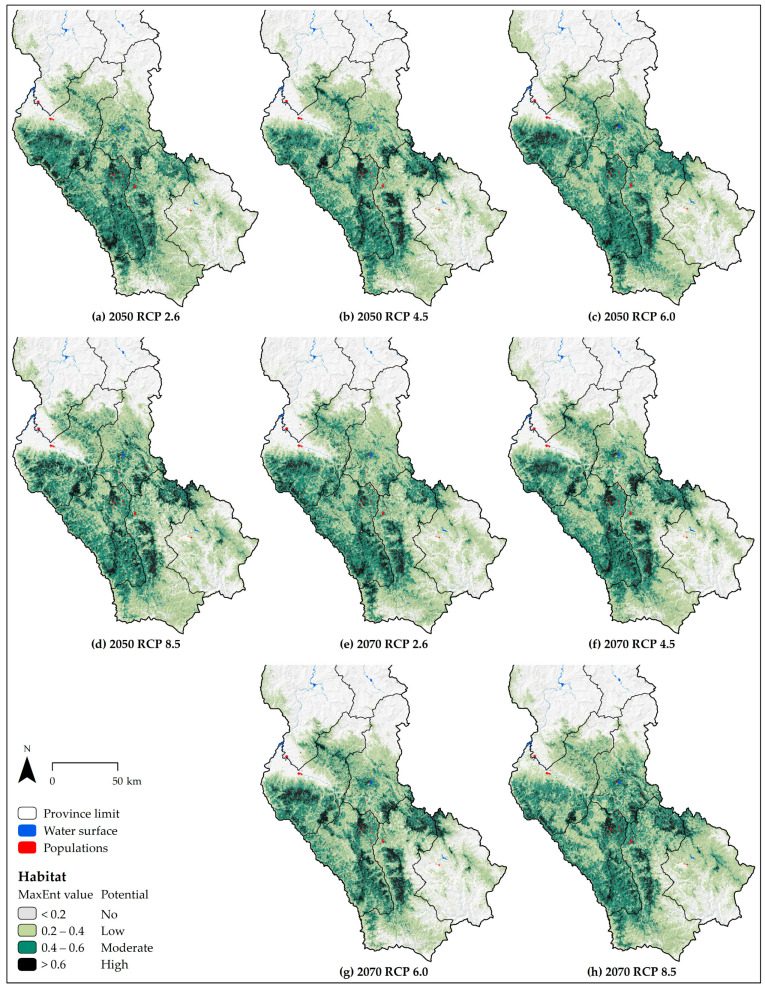
Potential distribution in climate change scenarios of spectacled bear in Amazonas (Peru).

Under current conditions, the area of total suitability including “high”, “moderate” and “low”potential habitat for potential distribution of the spectacled bear is modeled for the southwest of Amazonas (Figure 4). It is modelled in five (Bongará, Chachapoyas, Luya, Rodríguez de Mendoza, and Utcubamba) of the region’s seven provinces. In all climate change scenarios, the potential distribution shows a contraction, mainly from southeast to southwest Amazonas, across Rodríguez de Mendoza and Chachapoyas provinces (Figure 5). Areas of potential “high”, “moderate”, and “low” habitat under current conditions for the spectacled bear cover 1.99% (836.22 km^2^), 14.46% (6081.88 km^2^), and 20.73% (8718.98 km^2^) of Amazonas land, respectively (Table 3). Concerning these areas, “high” potential habitat will increase in all future scenarios, while “moderate” and “low” potential habitat will decrease. In sum, the area of total suitability, which covers 37.19% (15637.08 km^2^) of Amazonas land, will also decrease in all future scenarios. The “high” potential habitat will increase by a maximum of 65.1% (544.59 km^2^) by 2050 (at RCP 8.5) and 57.9% (484.47 km^2^) by 2070 (at RCP 4.5).

The IUCN expert map indicates that 14.31% (6018.68 km^2^) of Amazonas is in the “existing” zone for the presence of the spectacled bear (Figure 5). On the other hand, a figure which the model under current conditions predicted to be 16.45% (6918.10 km^2^) is under the combined “high” and “moderate” potential habitat range (>0.4 presence probability) and it reaches, moreover, 37.19% (15,637.08 km^2^) if the “low” potential habitat range (>0.2 presence probability) is also incorporated (Table 3). The predicted suitable areas are larger and cover about 75.31% (4513.94 km^2^) of the “existing” area of the IUCN map (Table 4), showing that there was a good overlap between both datasets. However, by reclassifying the “existing” area on the IUCN map into ranges, it covers about 34.3% (286.79 km^2^), 34.8% (2114.56 km^2^) and 24.2% (2112.60 km^2^) of the areas of potential distribution characterized with “high”, “moderate” and “low” habitat, respectively (Table 4). In addition, from overlapping the IUCN map with models in future scenarios and reclassification into ranges, it is determined that “high” potential habitat will increase in all future scenarios, meanwhile “moderate” potential (except 2050 RCP 6.0) and “low” potential habitat will decrease (Table 4). Overall, the total suitability area of the IUCN map, which covers 10.73% (4513.94 km^2^) of Amazonas land, is also projected to decrease in all future scenarios (except 2050 RCP 6.0).

### 3.3. Habitat Change and High-Priority Areas for Research and Conservation

Table 5 shows the area of total suitability (sum of “high”, “moderate” and “low” potential habitat) predicted for the spectacled bear according to the functional unit of the ecosystem it encompasses. The detail of the areas at the level of potential habitat ranges is presented in Table S2. Most, 81.2% (12,702.18 km^2^) of the total predicted suitability area, under current conditions, in Amazonas (15,637.08 km^2^; Table 3) is found in three functional ecosystem units. In largest to smallest habitat scale, 35.4% (5541.04 km^2^) is found in Yunga montane forest (B-mY), 24.6% in Secondary vegetation (PH and Vsec combined since PH is a sub-unit of Vsec, but was considered separately in Table 5 to improve information detail) and 21.1% (3306.78 km^2^) in Yunga altimontane forest (B-aY). Nevertheless, for the IUCN expert map (6018.68 km^2^) the order of the largest habitats is changed, the three previous ones reach 69.1% (4161.36 km^2^) and a fourth functional unit is required to surpass 80% of the current distribution. Namely, from largest to smallest habitat are, 26.5% (1594.54 km^2^) is in B-aY, 21.8% (1310.21 km^2^) in PH and Vsec combined, 20.9% (1256.61 km^2^) in B-mY and 19.6% (1179.97 km^2^) in Basimontano Yunga Forest (B-bY). On the other hand, although the total predicted area of suitability decreases in all climate change scenarios with respect to current conditions (Table 3). All future models retain the three main habitats (B-mY, PH and Vsec combined, B-aY) and the accumulated one close to >80% of the distribution (Table 5). At the level of potential distribution ranges, “moderate” and “low” potential habitats, under current conditions and climate change scenarios, are also distributed mainly in three functional units (B-mY, combined PH and Vsec, and B-aY). However, the “high” potential habitat leaves out B-mY and is mainly distributed over PH and Vsec combined, Jalca (Jal) and B-aY, indicating strong spectacled bear preferences for the Jal functional unit.

The importance of each functional unit is also determined by the percentage of its area that is occupied by the potential distribution of the spectacled bear. Thus, the total suitability predicted under current conditions occupies 99.1% of the total surface of the B-aY functional unit in Amazonas, 98.9% of the Andean Scrubland (Ma), 98.8% of the PH, 97.8% of the Jal, 81.5% of the Vsec, 69.4% of the B-mY, 67.9% of the Seasonally Dry Inter-Andean Forest (Bes-in), 29.9% of the Agricultural Zone (Agri) and less than 4% of the other two functional units (Table 5). On the other hand, the “existing” zone of the IUCN expert map occupies 49.9% of the total surface of the PH functional unit in Amazonas, 47.8% of B-aY, 43.3% of the Jal, 19.5% of the Vsec, 15.7% of the B-mY, 12.9 of the Ma, 7.2% of the Basimontano Yunga Forest (B-bY) and up to a maximum of 1.1% by other functional units (Table 5).

The predicted suitability areas were larger, and therefore, it covers a higher percentage of each functional unit compared to the “existing” area of the IUCN map. However, although, in general there are overlaps in the functional units that are most covered, there are serious differences in the functional units of Ma and Agri. The total adequacy predicted in all climate change scenarios occupies slightly less area of B-aY, Bes-in (except in 2070 RCP 8.5), B-mY, Jal, Ma, PH, Vsec and Agri (except in 2050 RCP 2.6 and 2070 RCP 8.5), whereas it occupies slightly more area of B-bY.

Table 6 shows the total potential distribution area (sum of habitats of “high”, “moderate” and “low” potential) predicted for the spectacled bear that was protected by the modalities of Protected Natural Area in the Amazon. The detail of the areas at the level of potential habitat ranges is presented in Table S2. In Amazonas, 15.4% (2407.19 km^2^) of the total suitability area predicted under current conditions (15,637.08 km^2^; Table 3) is covered by protected areas. This percentage drops to 10.6% if the ranges of “high” (88.48 km^2^) and “moderate” (642.29 km^2^) potential habitat are assessed separately. In fact, the 19 PCAs combined cover the largest percentage of predicted suitable areas for the spectacled bear with 9.1% (1427.39 km^2^). In contrast, 12.3% (740.49 km^2^) of the IUCN expert map in Amazonas (6018.68 km^2^) is covered by protected areas (Table 5), which means that the 19 PCAs combined (12.2%) followed by the two RCAs combined (0.1%) are the protection and conservation modalities. In all climate change scenarios, about 15% of the total predicted area of suitability is covered by protected areas. Furthermore, the 19 combined PCAs followed by the two combined RCAs cover the highest percentages of predicted suitable areas for the spectacled bear.

The importance of each type of NAP is also determined by the percentage of its area that is occupied by the potential distribution of the spectacled bear. Thus, the total suitability predicted under current conditions occupies 97.7% of the total area of the two RCA in Amazonas, 96.3% of the 19 PCA, 48.9% of the NS and less than 6% of the other three modalities (Table 5). The “existing” area of the IUCN expert map occupies 49.4% of the 19 PCAs and 1.2% of the two RCAs. The total adequacy predicted in all climate change scenarios occupies less area of the two RCAs (except in 2070 RCP 8.5) and 19 ACs, while it occupies more area of the NS.

## 4. Discussion

Our study is the first study that contributes the future distribution of the spectacled bear and it sends alarms indicating the decline in environmentally suitable areas for the species.

### 4.1. Variables and the Performance of the Models

We selected 12 of 23 environmental variables for modeling of the potential distribution of spectacled bears. The mean temperature of driest quarter (bio09), precipitation of driest month (bio14) and percentage of forest cover (shelter availability) drove up to 75.2% of the potential distribution in our models. Figueroa et al. [17] only used 8 of the 12 variables used by us, and the seasonality of precipitation (bio15, 61.7%), followed by isothermality (bio03, 15.7%) and bio09 (10.2%) were the variables with the greatest contribution to its model in the regions of Amazonas and Cajamarca. Although bio09 is also considered one of the most important, bio15 only contributed 13.7% in current conditions and <5% in future scenarios (except 2050 RCP 6.0, 8.3%) in our models. The different methods used to select the variables and, therefore, the different variables introduced in the models, contributed to differentiate their contribution. The slope variable had no contribution to the model (0%) in Figueroa et al. [17], and in our models slope also have low contributions (3.7–5.8%). This may be because bears are not limited by steep slopes, where they even forage [4]. The percentage of forest cover (availability of shelter) was one of the three significant variables that provides high contributing to the distribution models (18–25%). Among different variables, for the model of spectacled bears in Bolivia [28], the availability of shelter was the variable that have highest contribution (31.8%). The biological relevance of the refuge could be explained by the extensive arboreal activity of spectacled bears, including the construction of nests or platforms and the feeding of fruits, epiphytic bromeliads and orchid pseudobulbs [60].

Generated nine models had excellent predictive performances (AUC > 0.9). These performances are higher than those obtained for spectacled bear models in Bolivia (0.823 and 0.851) [28], and are equal to or more accurate than those reported for models of other bear species (*Helarctos malayanus* [18], *Ursus arctos* [24], *Ursus thibetanus ussuricus* [25], *Ursus thibetanus gedrosianus* [27], and *Ursus thibetanus* [29]).

### 4.2. Distribution and Changes of Habitat

Under current conditions, the area of total suitability including “high”, “moderate” and “low” potential habitat of the spectacled bear was modeled to the southwest of Amazonas. Only the potential “high” habitat presented ranges of mean annual temperature, annual accumulated precipitation and elevation of 10.1–22.6 °C, 402–1502 mm and 470–3700 m a.s.l. Most, 81.2% of the total suitability area predicted was concentrated in the Yunga montane forest (35.4%), combined Grassland/Herbaceous and Secondary Vegetation (24.6%) and the Yunga altimontane (rain) forest (21.1%). The “moderate” and “low” potential habitats are also mainly distributed in these three habitats, while “high” potential habitat was also concentrated in the Jalca. In brief, the total suitability predicted under current conditions occupies 81.5% to 99.1% of the surface area of the aforementioned habitats, including the Andean scrubland. These percentages vary slightly in climate change scenarios. Another important ecosystem that spectacled bears inhabit in the Amazonas were the inter-Andean dry forests, distributed in 67.9% of this ecosystem. Figueroa et al. [2,5,17] has carried out extensive studies on the species and this xeric ecosystem with a high degree of diversity and endemism. The wide distribution of the spectacled bear is well documented [1,4,60] and is a property that contributes to its being an umbrella species of other species [5]. A recent study indicates that 20.6% of the area of the 12 Andean ecoregions that it inhabits is covered by the range of the spectacled bear, and the total number of vertebrate and threatened species is higher in areas where bears are present than in those in which they are absent [7]. Likewise, its mobility allows it to be a legitimate disperser of the seeds of some fruits it consumes, for which it is attributed an important role in the recovery and regeneration of forests [5].

The high percentages of predicted total suitability areas that coincide with the functional unit of secondary vegetation (PH and Vsec combined) indicate that spectacled bear habitats have suffered severe by anthropogenic disturbance. These areas include grasslands that have been cleared and converted to cultivated pasture, as well as areas covered with secondary vegetation (“purma”) in the Amazon, which are resting for a certain number of years until the natural fertility of the soil returns, thus being re-integrated into agricultural activity [30]. In fact, the Peruvian Amazon has lost 22,848.88 km^2^ of forest between 2001 and 2018, and Amazonas ranks eighth with 880 km^2^ of forest loss [61]. Studies in Amazonas [62], indicate that deforestation has maintained increasing rates in recent years. Figueroa et al. [17] highlights that the loss of the habitat of the spectacled bear is due to the construction of road and hydroelectric networks in the dry forest of the Marañón (border between Amazonas and Cajamarca). The road networks allow the trade of products from the area, however, they also facilitate the entry of hunters and loggers, and also the fragmentation of forests due to livestock and crops. Due to such conditions bear-human conflicts have increased, which motivated the establishment of guidelines for the management of these conflicts [9].

The adequate area predicted by our model under current conditions is larger than the area of the IUCN expert map [11]. The IUCN map presents large areas on the western border of Amazonas with Cajamarca region (in Bagua and Condorcanqui provinces) that may be probable habitats, but have not been identified as suitable in our model. One possible reason could be the limited number of records of the presence of the spectacled bear that we use in the aforementioned area [18]. Conversely, our model shows large areas with “high” and “moderate” potential habitat ranges located in Utcubamba province (in the Cajaruro district and on the southwestern border of this province), the border of Bongará, Chachapoyas and Luya provinces, and the northwestern border of Luya province, north of Rodríguez de Mendoza province (in Vista Alegre district) and in Bongará province (mostly in Florida, Quisquilla and Jumbilla districts). These locations were not marked as “existing” on the IUCN map, despite having definitive sighting records from this area. Namely, only 32 of the 92 georeferenced records of the spectacled bear used for our model are within the IUCN map. In addition, this IUCN map excludes an area with important scientific reports of spectacled bears in Amazonas, such as Corosha [63] and the upper basin of the Gocta waterfall [64].

Although “high” potential habitat will increase in all climate change scenarios, “moderate” and “low” potential habitats, as well as overall suitability, will decrease. However, it should be considered that biogeographic simulations in climate change scenarios should be interpreted with caution, since they can overestimate the decline or increase, by not considering the qualities of the species to adapt in situ to new conditions, or persist outside of the conditions in which they have been observed [65,66]. It is unknown the adaptation of the socio-ecological system to future climate scenarios, particularly for those organisms intrinsically linked to forest health and seed dispersal [67].

### 4.3. Conservation of the Spectacled Bear

In Peru, the main strategy for in situ conservation is the establishment of public and private NPAs [68]. Namely, Amazonas is one of the regions that registers the most NPAs [15]. Figueroa [2] indicates that the presence of the Andean bear has been registered in 55 of the 76 Peru’s NAPs, but that it is unknown how much the legal protection of the habitat (in km^2^) of the species amounts to. The low percentages of total suitability areas predicted, which are covered by the NPA system in Amazonas, are indicators that the system is not as extensive or precise as it should be to cover most of the important habitats for the spectacled bear. We observe that NPAs are not established considering potential distribution maps (based on SDM) of the species that are intended to be conserved. In that sense, it is precise to recommend that the establishment of future protected areas use as one of the inputs, the geographic limits predicted by an MDS, for a species or species intended to be conserved and protected [69]. Furthermore, the models projected in climate change scenarios could help more in this task, because even if there are changes in the distribution of the species, the relatively stable distribution sites will be considered with maximum interest.

The need to conserve spectacled bears has led to ecoregional [8] and multinational [6] commitments. In Peru, the National Plan for the Conservation of the Andean Bear (*Tremarctos ornatus*) was implemented for the period 2016–2026 [70]. The main objective is to recover spectacled bear populations through conservation measures for the species and its habitats, and one of its activities is the determination of the current and historical distribution areas of the species. This study provides key information for the national plan and future conservation initiatives, identifying areas of interest for the spectacled bear. This methodology can be applied to other key species and at the national level, with the necessary complements even for other ecosystems in the world.

## 5. Conclusions

Areas of “high”, “moderate” and “low” potential habitat under current conditions for the spectacled bear cover 1.99% (836.22 km^2^), 14.46% (6081.88 km^2^) and 20.73% (8718.98 km^2^) of Amazonas land area, respectively. “High” potential habitat is expected to increase under all climate change scenarios. The “moderate” and “low” potential habitats, as well as overall suitability, are estimated to decrease over time. The comparison of the predicted model with the IUCN expert based map showed that there are differences in distribution zones and area in Amazonas region. In addition, comparison of the predicted model with the map of Natural Protected Areas indicates that these areas are not large enough to cover most of the important habitats for the spectacled bear, both under current conditions and scenarios of climate change. In fact, to effectively conserve spectacled bears, the “high” (even “moderate”) potential habitat identified in predictive modelling, the areas on the IUCN map where spectacled bears occur, and the main ecosystems that the species inhabits, it is recommended to convert them into priority areas for research and conservation (even convert them into natural protected areas). Ultimately, the model provides inputs for a better understanding of spectacled bear distribution and habitat preferences in the Amazonas region, that provides key information for the national conservation plan of the species, to design surveys to locate new individuals for the future conservation and management plans.

## Figures and Tables

**Table 1:** Performance of MaxEnt models of *T. ornatus* in Amazonas (Peru).

Performance	Current	2050				2070			
		RCP 2.6	RCP 4.5	RCP 6.0	RCP 8.5	RCP 2.6	RCP 4.5	RCP 6.0	RCP 8.5
AUC	0.907	0.909	0.913	0.908	0.903	0.909	0.907	0.915	0.905
Std Dev	0.014	0.014	0.012	0.011	0.008	0.007	0.011	0.012	0.014

**Table 2:** Relative contributions (%) of environmental variables to the MaxEnt model under current conditions and climate change scenarios of the spectacled bear in Amazonas (Peru).

Variables	Current	2050				2070			
		RCP 2.6	RCP 4.5	RCP 6.0	RCP 8.5	RCP 2.6	RCP 4.5	RCP 6.0	RCP 8.5
bio02	1.5	4.0	2.5	2.3	1.9	3.3	2.6	4.4	1.9
bio03	1.7	4.6	3.8	2.6	1.8	2.4	2.3	2.3	0.6
bio04	0.8	2.0	1.6	0.8	2.4	1.7	0.5	0.7	2.0
bio07	5.1	5.9	5.2	6.8	2.2	6.4	2.2	3.7	3.6
bio09	33.3	14.2	21.7	14.0	22.6	15.3	22.7	25.4	20.5
bio12	3.1	2.9	2.9	3.1	2.3	2.8	3.4	2.7	2.9
bio14	14.6	31.0	26.8	36.5	28.4	37.7	27.3	22.9	30.6
bio15	13.7	2.4	2.6	8.3	2.6	3.9	4.7	4.2	3.1
Elevation	2.2	1.9	4.5	1.1	8.6	1.2	2.6	2.0	2.1
Slope	3.7	3.8	5.3	3.9	4.2	6.2	5.0	4.4	5.8
Water	1.6	2.4	2.2	1.8	1.5	2.1	2.3	3.1	2.8
Forest	18.8	25.0	20.9	18.8	21.5	18.0	24.4	24.3	24.1
Total (%)	100.0	100.0	100.0	100.0	100.0	100.0	100.0	100.0	100.0

**Table 3:** Potential predicted distribution area (km^2^) under current conditions and variation (%) in climate change scenarios of the spectacled bear in Amazonas (Peru).

Habitat Potential	Current (km^2^)	2050 ^1^				2070 (%) ^1,2^			
		RCP 2.6	RCP 4.5	RCP 6.0	RCP 8.5	RCP 2.6	RCP 4.5	RCP 6.0	RCP 8.5
High	836.22	1291.78	1182.01	1110.70	1380.81	1241.46	1320.69	1313.54	1228.92
		*54.5*	*41.4*	*32.8*	*65.1*	*48.5* *(−3.9)*	*57.9* *(11.7)*	*57.1* *(18.3)*	*47.0* *(−11.0)*
Moderate	6081.88	5138.23	4841.07	5636.84	5237.86	5108.22	5007.23	4479.66	5648.26
		*−15.5*	*−20.4*	*−7.3*	*−13.9*	*−16.0* *(−0.6)*	*−17.7* *(3.4)*	*−26.3* *(−20.5)*	*−7.1* *(7.8)*
Low	8718.98	8090.52	7886.08	7978.52	8520.09	8114.29	7808.02	7584.25	8435.83
		*−7.2*	*−9.6*	*−8.5*	*−2.3*	*−6.9* *(0.3)*	*−10.4* *(−1.0)*	*−13.0* *(−4.9)*	*−3.2* *(−1.0)*
Total	15637.08	14520.53	13909.16	14726.06	15138.76	14463.97	14135.94	13377.45	15313.01
		*−7.1*	*−11.1*	*−5.8*	*−3.2*	*−7.5* *(−0.4)*	*−9.6* *(1.6)*	*−14.5* *(−9.2)*	*−2.1* *(1.2)*

^1^ The area in km^2^ in normal font and the variation (%) from current conditions in italics. ^2^ In parenthesis, the change (%) from the same RCP in 2050.

**Table 4:** Area (km^2^) of the “existing” land area on the IUCN map that is indexed to the predicted potential distribution under current conditions and variation (%) in climate change scenarios of the spectacled bear in Amazonas (Peru).

Habitat Potential	Current IUCN (km^2^)	2050 ^1^				2070 ^1,2^			
		RCP 2.6	RCP 4.5	RCP 6.0	RCP 8.5	RCP 2.6	RCP 4.5	RCP 6.0	RCP 8.5
High	286.79	643.17	639.60	544.85	616.40	598.75	687.07	608.98	541.70
		*124.3*	*123.0*	*90.0*	*114.9*	*108.8* *(−6.9)*	*139.6* *(7.4)*	*112.3* *(11.8)*	*88.9* *(−12.1)*
Moderate	2114.56	2005.35	1836.56	2148.76	1820.37	1947.08	1943.70	1554.85	1861.10
		*−5.2*	*−13.1*	*1.6*	*−13.9*	*−7.9* *(−2.9)*	*−8.1* *(5.8)*	*−26.5* *(−27.6)*	*−12.0* *(2.2)*
Low	2112.60	1727.78	1773.77	1894.49	1935.98	1720.07	1800.52	1855.32	1905.27
		*−18.2*	*−16.0*	*−10.3*	*−8.4*	*−18.6* *(−0.4)*	*−14.8* *(1.5)*	*−12.2* *(−2.1)*	*−9.8* *(−1.6)*
Total	4513.94	4376.31	4249.93	4588.10	4372.75	4265.89	4431.29	4019.15	4308.06
		*−3.0*	*−5.8*	*1.6*	*−3.1*	*−5.5* *(−2.5)*	*−1.8* *(4.3)*	*−11.0* *(−12.4)*	*−4.6* *(−1.5)*

^1^ The area in km^2^ in normal font and the variation (%) from current conditions in italics. ^2^ In parenthesis, the change (%) from the same CPR in 2050.

**Table 5:** Area (in km^2^ and %) of the total potential distribution predicted both in current conditions and in climate change scenarios of the spectacled bear according to the functional unit of the ecosystem it encompasses in Amazonas (Peru).

Functional Units of the Ecosystem ^1^	Current	2050 ^2^				2070 ^2^				IUCN Extant
		RCP 2.6	RCP 4.5	RCP 6.0	RCP 8.5	RCP 2.6	RCP 4.5	RCP 6.0	RCP 8.5	
B-aY	3306.78	3131.59	3152.04	3249.92	3150.87	3154.37	3220.79	3017.18	3247.13	1594.54
	*21.0* *(99.1)*	*21.6* *(93.8)*	*22.7* *(94.4)*	*22.1 (97.4)*	*20.8 (94.4)*	*21.8* *(94.5)*	*22.8* *(96.5)*	*22.6* *(90.4)*	*21.2* *(97.3)*	*26.5* *(47.8)*
B-bY	434.22	531.55	490.01	468.71	519.33	488.27	478.62	515.68	488.00	1179.97
	*2.8* *(2.6)*	*3.7* *(3.2)*	*3.5* *(3.0)*	*3.2* *(2.8)*	*3.4* *(3.2)*	*3.4* *(3)*	*3.4* *(2.9)*	*3.9* *(3.1)*	*3.2* *(3.0)*	*19.6* *(7.2)*
Bes-in	635.97	547.94	529.65	560.46	468.76	513.78	553.37	498.85	662.00	3.09
	*4.1* *(67.9)*	*3.8* *(58.5)*	*3.8* *(56.6)*	*3.8* *(59.9)*	*3.1* *(50.1)*	*3.6* *(54.9)*	*3.9* *(59.1)*	*3.7* *(53.3)*	*4.3* *(70.7)*	*0.1* *(0.3)*
B-mY	5541.04	4880.56	4340.57	4916.05	5466.58	4927.62	4467.73	4132.46	5201.75	1256.61
	*35.4* *(69.4)*	*33.6* *(61.1)*	*31.2* *(54.3)*	*33.4* *(61.5)*	*36.1* *(68.4)*	*34.1* *(61.7)*	*31.6* *(55.9)*	*30.9* *(51.7)*	*34.0* *(65.1)*	*20.9* *(15.7)*
Jal	1407.49	1235.26	1302.16	1374.86	1373.31	1292.78	1313.94	1243.68	1369.05	623.52
	*9.0* *(97.8)*	*8.5* *(85.8)*	*9.4* *(90.5)*	*9.3* *(95.5)*	*9.1* *(95.4)*	*8.9* *(89.8)*	*9.3* *(91.3)*	*9.3* *(86.4)*	*8.9* *(95.1)*	*10.4* *(43.3)*
Ma	321.86	312.39	318.21	320.11	310.82	319.06	319.19	309.73	319.45	41.93
	*2.1* *(98.9)*	*2.2* *(96.0)*	*2.3* *(97.8)*	*2.2* *(98.4)*	*2.1* *(95.5)*	*2.2* *(98)*	*2.3* *(98.1)*	*2.3* *(95.2)*	*2.1* *(98.2)*	*0.7* *(12.9)*
PH	1460.26	1453.60	1442.36	1456.18	1444.68	1442.99	1444.52	1412.13	1458.54	737.57
	*9.3* *(98.8)*	*10.0* *(98.3)*	*10.4* *(97.5)*	*9.9* *(98.5)*	*9.5* *(97.7)*	*10.0* *(97.6)*	*10.2* *(97.7)*	*10.6* *(95.5)*	*9.5* *(98.6)*	*12.3* *(49.9)*
Vsec	2394.09	2289.94	2232.60	2274.34	2320.23	2221.65	2236.51	2161.23	2380.32	572.64
	*15.3* *(81.5)*	*15.8* *(78.0)*	*16.1* *(76.0)*	*15.4* *(77.5)*	*15.3* *(79)*	*15.4* *(75.7)*	*15.8* *(76.2)*	*16.2* *(73.6)*	*15.5* *(81.1)*	*9.5* *(19.5)*
Agri	117.25	119.61	87.06	91.22	70.60	88.60	86.69	72.81	168.23	3.76
	*0.7* *(29.9)*	*0.8* *(30.5)*	*0.6* *(22.2)*	*0.6* *(23.2)*	*0.5* *(18.0)*	*0.6* *(22.6)*	*0.6* *(22.1)*	*0.5* *(18.5)*	*1.1* *(42.9)*	*0.1* *(1.0)*
Others	18.13	18.10	14.49	14.20	13.58	14.84	14.58	13.70	18.54	5.06
	*0.7* *(3.9)*	*0.8* *(3.9)*	*0.6* *(3.1)*	*0.6* *(3.1)*	*0.5* *(2.9)*	*0.6* *(3.2)*	*0.6* *(3.2)*	*0.5* *(3.0)*	*1.1* *(4.0)*	*0.1* *(1.1)*

^1^ B-aY: Yunga Altimontane (Pluvial) Forest, B-bY: Yunga Basimontane Forest, Bes-in: Seasonally dry inter-Andean forest, B-mY: Yunga mountain forest, Jal: Jalca, Ma: Andean scrub, PH: Grasslands/Herbazales, Vsec: Secondary Vegetation, Agri: Agricultural Zone, and others. ^2^ The area in km^2^ in normal font and its meaning in percentage (%) in italics. Without parentheses the percentage (%) with respect to the area of the potential habitat range and in parentheses the percentage (%) with respect to the area of the functional unit of the ecosystem.

**Table 6:** Area (in km^2^ and %) of the total potential distribution predicted both in current conditions and in climate change scenarios of the spectacled bear that was protected by the modalities of Protected Natural Area in Amazonas (Peru).

NPA Modalities ^1^	Current	2050 ^2^				2070 ^2^				IUCN Extant
		RCP 2.6	RCP 4.5	RCP 6.0	RCP 8.5	RCP 2.6	RCP 4.5	RCP 6.0	RCP 8.5	
NP	29.65	80.27	2.57	0.74	0.96	0.07	24.02	31.30	4.17	0.00
	*0.2* *(3.4)*	*0.6* *(9.1)*	*0.0* *(0.3)*	*0.0* *(0.1)*	*0.0* *(0.1)*	*0.0* *(0.0)*	*0.2* *(2.7)*	*0.2* *(3.5)*	*0.0* *(0.5)*	*0.0* *(0.0)*
NS	191.69	243.83	249.25	219.59	255.48	254.85	213.52	241.29	245.68	0.00
	*1.2* *(48.9)*	*1.7* *(62.2)*	*1.8* *(63.6)*	*1.5* *(56.0)*	*1.7* *(65.1)*	*1.8* *(65.0)*	*1.5* *(54.4)*	*1.8* *(61.5)*	*1.6* *(62.6)*	*0.0* *(0.0)*
CR	67.61	135.35	134.45	101.49	131.45	127.90	96.36	114.52	117.63	0.00
	*0.4* *(5.7)*	*0.9* *(11.4)*	*1.0* *(11.3)*	*0.7* *(8.6)*	*0.9* *(11.1)*	*0.9* *(10.8)*	*0.7* *(8.1)*	*0.9* *(9.7)*	*0.8* *(9.9)*	*0.0* *(0.0)*
RZ	76.63	67.53	74.65	69.23	143.22	114.87	46.00	76.07	115.33	0.00
	*0.5* *(5.5)*	*0.5* *(4.8)*	*0.5* *(5.3)*	*0.5* *(4.9)*	*0.9* *(10.2)*	*0.8* *(8.2)*	*0.3* *(3.3)*	*0.6* *(5.4)*	*0.8* *(8.2)*	*0.0* *(0.0)*
RCA	614.22	435.32	423.55	481.38	563.93	503.77	498.47	444.49	619.13	7.86
	*3.9* *(97.7)*	*3.0* *(69.2)*	*3.0* *(67.4)*	*3.3* *(76.6)*	*3.7* *(89.7)*	*3.5* *(80.1)*	*3.5* *(79.3)*	*3.3* *(70.7)*	*4.0* *(98.5)*	*0.1* *(1.2)*
PCA	1427.39	1258.53	1281.45	1385.11	1344.08	1300.19	1300.85	1126.94	1321.21	732.63
	*9.1* *(96.3)*	*8.7* *(84.9)*	*9.2* *(86.4)*	*9.4* *(93.4)*	*8.9* *(90.7)*	*9.0* *(87.7)*	*9.2* *(87.8)*	*8.4* *(76.0)*	*8.6* *(89.1)*	*12.2* *(49.4)*
Total	2407.19	2220.83	2165.93	2257.53	2439.12	2301.66	2179.21	2034.62	2423.15	740.49
	*15.4* *(40.3)*	*15.3* *(37.2)*	*15.6* *(36.2)*	*15.3* *(37.8)*	*16.1* *(40.8)*	*15.9* *(38.5)*	*15.4* *(36.5)*	*15.2* *(34.0)*	*15.8* *(40.5)*	*12.3* *(12.4)*

^1^ NP: National Park, NS: National Sanctuary, CR: Communal Reserves, RZ: Reserved Areas, RCA: Regional Conservation Areas, PCA: Private Conservation Areas. ^2^ The area in km^2^ in normal font and its meaning in percentage (%) in italics. Without parentheses the percentage (%) with respect to the area of the potential habitat range and in parentheses the percentage (%) with respect to the area of the NPA modalities.

## Supplementary Materials

The following are available online at https://www.mdpi.com/2076-2615/10/10/1816/s1. Table S1. Pearson’s correlation coefficients (r) between the bioclimatic variables for the modeling of the potential distribution of *T. ornatus* in Amazonas (Peru). Table S2. Area (in km^2^ and %) of the total potential distribution predicted both in current conditions and in the climate change scenarios of the spectacled bear according to the functional unit of the ecosystem it encompasses and the area that is protected by the Natural Area modalities Protected in Amazonas (Peru). Figure S1. (a) Optimal number of clusters and (b) Cluster dendrogram between the bioclimatic variables for the modeling of the potential distribution of *T. ornatus* a in Amazonas (Peru). Figure S2. Jackknife test in a preliminary model generated using only the 19 bioclimatic variables for the modeling of the potential distribution of *T. ornatus* a in Amazonas (Peru).
