# Supplementary material for: Predictive Modelling of Current and Future Potential Distribution of the Spectacled Bear (Tremarctos ornatus) in Amazonas, Northeast Peru
Source: Animals (Basel). 2020 Oct 6;10(10):1816. doi: 10.3390/ani10101816 (PMC7650621; doi:10.3390/ani10101816)
Supplement: Supplementary file 1 [file animals-10-01816-s001.docx]

Supplementary Materials

Predictive modelling of current and future potential distribution of the spectacled bear (*Tremarctos ornatus*) in Amazonas, northeast Peru

Gerson Meza Mori ^1, *^, Elgar Barboza Castillo ^1^, Cristóbal Torres Guzmán ^1^, Dany A. Cotrina Sánchez ^1^, Betty K. Guzman Valqui ^1^, Manuel Oliva ^1^, Subhajit Bandopadhyay ^2^, Rolando Salas López ^1^ and Nilton B. Rojas Briceño ^1, *^

^1^ Instituto de Investigación para el Desarrollo Sustentable de Ceja de Selva (INDES-CES), Universidad Nacional Toribio Rodríguez de Mendoza de Amazonas (UNTRM), Chachapoyas 01001, Perú; [ebarboza@indes-ces.edu.pe](mailto:ebarboza@indes-ces.edu.pe) (E.B.C.); [cristobal.torres@untrm.edu.pe](mailto:cristobal.torres@untrm.edu.pe) (C.T.G.); alexander.cotrina@untrm.edu.pe (D.A.C.S.); betty.guzman@untrm.edu.pe (B.K.G.V.), [soliva@indes-ces.edu.pe](mailto:soliva@indes-ces.edu.pe) (M.O.); [rsalas@indes-ces.edu.pe](mailto:rsalas@indes-ces.edu.pe) (R.S.L.)

^2^ Department of Ecology and Environmental Protection; Poznan University of Life Sciences; Piatkowska 94; 60-649 Poznan; Poland; [subhajit.bandopadhyay@up.poznan.pl](mailto:subhajit.bandopadhyay@up.poznan.pl) (S.B.)

***** Correspondence: [gmeza@indes-ces.edu.pe](mailto:gmori@indes-ces.edu.pe); Tel.: +51-996-907-768; [nrojas@indes-ces.edu.pe](mailto:nrojas@indes-ces.edu.pe) (N.B.R.B.); Tel.: +51-949-667-638

Received: date; Accepted: date; Published: date

**Table S1.** Pearson's correlation coefficients (r) between the bioclimatic variables for the modelling of the potential distribution of *T. ornatus* in Amazonas (Peru).

|  | bio01 | bio02 | bio03 | bio04 | bio05 | bio06 | bio07 | bio08 | bio09 | bio10 | bio11 | bio12 | bio13 | bio14 | bio15 | bio16 | bio17 | bio18 | bio19 |
| --- | --- | --- | --- | --- | --- | --- | --- | --- | --- | --- | --- | --- | --- | --- | --- | --- | --- | --- | --- |
| bio01 | 1 | -0.309 | -0.665 | 0.594 | 0.990 | 0.989 | -0.065 | 0.999 | 1.000 | 0.999 | 1.000 | -0.909 | -0.938 | -0.681 | 0.209 | -0.925 | -0.662 | -0.906 | -0.643 |
| bio02 | -0.309 | 1 | 0.628 | -0.021 | -0.180 | -0.437 | 0.933 | -0.306 | -0.315 | -0.300 | -0.315 | 0.302 | 0.457 | 0.061 | 0.261 | 0.445 | 0.044 | 0.429 | 0.040 |
| bio03 | -0.665 | 0.628 | 1 | -0.490 | -0.622 | -0.695 | 0.306 | -0.666 | -0.662 | -0.667 | -0.662 | 0.560 | 0.750 | 0.186 | 0.303 | 0.713 | 0.181 | 0.647 | 0.168 |
| bio04 | 0.594 | -0.021 | -0.490 | 1 | 0.621 | 0.551 | 0.209 | 0.613 | 0.574 | 0.618 | 0.572 | -0.558 | -0.501 | -0.410 | 0.247 | -0.517 | -0.395 | -0.402 | -0.372 |
| bio05 | 0.990 | -0.180 | -0.622 | 0.621 | 1 | 0.960 | 0.072 | 0.990 | 0.989 | 0.992 | 0.989 | -0.891 | -0.907 | -0.679 | 0.232 | -0.894 | -0.662 | -0.874 | -0.643 |
| bio06 | 0.989 | -0.437 | -0.695 | 0.551 | 0.960 | 1 | -0.209 | 0.988 | 0.990 | 0.987 | 0.990 | -0.903 | -0.947 | -0.660 | 0.176 | -0.935 | -0.640 | -0.919 | -0.621 |
| bio07 | -0.065 | 0.933 | 0.306 | 0.209 | 0.072 | -0.209 | 1 | -0.062 | -0.075 | -0.054 | -0.074 | 0.105 | 0.209 | -0.018 | 0.183 | 0.210 | -0.035 | 0.223 | -0.034 |
| bio08 | 0.999 | -0.306 | -0.666 | 0.613 | 0.990 | 0.988 | -0.062 | 1 | 0.998 | 0.999 | 0.998 | -0.912 | -0.938 | -0.686 | 0.217 | -0.927 | -0.667 | -0.902 | -0.648 |
| bio09 | 1.000 | -0.315 | -0.662 | 0.574 | 0.989 | 0.990 | -0.075 | 0.998 | 1 | 0.998 | 1.000 | -0.907 | -0.939 | -0.678 | 0.204 | -0.925 | -0.659 | -0.909 | -0.640 |
| bio10 | 0.999 | -0.300 | -0.667 | 0.618 | 0.992 | 0.987 | -0.054 | 0.999 | 0.998 | 1 | 0.998 | -0.908 | -0.934 | -0.679 | 0.215 | -0.922 | -0.661 | -0.900 | -0.641 |
| bio11 | 1.000 | -0.315 | -0.662 | 0.572 | 0.989 | 0.990 | -0.074 | 0.998 | 1.000 | 0.998 | 1 | -0.908 | -0.940 | -0.679 | 0.205 | -0.926 | -0.661 | -0.910 | -0.642 |
| bio12 | -0.909 | 0.302 | 0.560 | -0.558 | -0.891 | -0.903 | 0.105 | -0.912 | -0.907 | -0.908 | -0.908 | 1 | 0.919 | 0.870 | -0.454 | 0.954 | 0.861 | 0.932 | 0.853 |
| bio13 | -0.938 | 0.457 | 0.750 | -0.501 | -0.907 | -0.947 | 0.209 | -0.938 | -0.939 | -0.934 | -0.940 | 0.919 | 1 | 0.638 | -0.078 | 0.990 | 0.617 | 0.954 | 0.607 |
| bio14 | -0.681 | 0.061 | 0.186 | -0.410 | -0.679 | -0.660 | -0.018 | -0.686 | -0.678 | -0.679 | -0.679 | 0.870 | 0.638 | 1 | -0.787 | 0.700 | 0.993 | 0.703 | 0.991 |
| bio15 | 0.209 | 0.261 | 0.303 | 0.247 | 0.232 | 0.176 | 0.183 | 0.217 | 0.204 | 0.215 | 0.205 | -0.454 | -0.078 | -0.787 | 1 | -0.172 | -0.807 | -0.210 | -0.807 |
| bio16 | -0.925 | 0.445 | 0.713 | -0.517 | -0.894 | -0.935 | 0.210 | -0.927 | -0.925 | -0.922 | -0.926 | 0.954 | 0.990 | 0.700 | -0.172 | 1 | 0.684 | 0.964 | 0.676 |
| bio17 | -0.662 | 0.044 | 0.181 | -0.395 | -0.662 | -0.640 | -0.035 | -0.667 | -0.659 | -0.661 | -0.661 | 0.861 | 0.617 | 0.993 | -0.807 | 0.684 | 1 | 0.690 | 0.999 |
| bio18 | -0.906 | 0.429 | 0.647 | -0.402 | -0.874 | -0.919 | 0.223 | -0.902 | -0.909 | -0.900 | -0.910 | 0.932 | 0.954 | 0.703 | -0.210 | 0.964 | 0.690 | 1 | 0.684 |
| bio19 | -0.643 | 0.040 | 0.168 | -0.372 | -0.643 | -0.621 | -0.034 | -0.648 | -0.640 | -0.641 | -0.642 | 0.853 | 0.607 | 0.991 | -0.807 | 0.676 | 0.999 | 0.684 | 1 |

**Table S2.** Area (in km^2^ and %) of the total potential distribution predicted both in current conditions and in the climate change scenarios of the spectacled bear according to the functional unit of the ecosystem it encompasses and the area that is protected by the Natural Area modalities Protected in Amazonas (Peru).

| **Model** | **Habitat potential** | **Area**  **(km^2^)** | **Functional units of the ecosystem ^1, 2^** | | | | | | | | | |  | **Protected natural area modalities ^1, 3^** | | | | | | |
| --- | --- | --- | --- | --- | --- | --- | --- | --- | --- | --- | --- | --- | --- | --- | --- | --- | --- | --- | --- | --- |
|  |  |  | **B-aY** | **B-bY** | **Bes-in** | **B-mY** | **Jal** | **Ma** | **PH** | **Vsec** | **Agri** | **Others** |  | **NP** | **NS** | **CR** | **RZ** | **RCA** | **PCA** | **Total** |
|  |  |  | 3337.79 | 16455.08 | 936.38 | 7988.75 | 1439.05 | 325.42 | 1478.62 | 2936.23 | 392.59 | 461.36 |  | 884.77 | 392.16 | 1185.65 | 1403.37 | 628.74 | 1482.34 | 5977.03 |
| Current | High | 836.22 | 105.14  *12.6 (3.1)* | 7.35  0.9 (0) | 44.28  *5.3 (4.7)* | 71.39  *8.5 (0.9)* | 237.35  *28.4 (16.5)* | 37.19  *4.4 (11.4)* | 140.07  *16.8 (9.5)* | 191.15  *22.9 (6.5)* | 0.37  *0 (0.1)* | 1.93  *0 (0.4)* |  | 0.00  *0 (0)* | 19.08  *2.3 (4.9)* | 6.25  *0.7 (0.5)* | 0.00  *0 (0)* | 23.25  *2.8 (3.7)* | 39.90  *4.8 (2.7)* | 88.48  *10.6 (1.5)* |
|  | Moderate | 6081.88 | 1281.59  *21.1 (38.4*) | 119.54  *2.0 (0.7)* | 259.56  *4.3 (27.7)* | 1193.70  *19.6 (14.9)* | 815.68  *13.4 (56.7)* | 235.39  *3.9 (72.3)* | 889.68  *14.6 (60.2)* | 1262.38  *20.8 (43.0)* | 18.10  *0.3 (4.6)* | 6.27  *0.3 (1.4)* |  | 0.01  *0 (0)* | 28.21  *0.5 (7.2)* | 6.38  *0.1 (0.5)* | 0.34  *0 (0)* | 193.31  *3.2 (30.7)* | 414.04  *6.8 (27.9)* | 642.29  *10.6 (10.7)* |
|  | Low | 8718.98 | 1920.05  *22 (57.5)* | 307.33  *3.5 (1.9)* | 332.13  *3.8 (35.5)* | 4275.95  *49.0 (53.5)* | 354.46  *4.1 (24.6)* | 49.28  *0.6 (15.1)* | 430.51  *4.9 (29.1)* | 940.56  *10.8 (32.0)* | 98.78  *1.1 (25.2)* | 9.92  *1.1 (2.2)* |  | 29.64  *0.3 (3.3)* | 144.39  *1.7 (36.8)* | 54.98  *0.6 (4.6)* | 76.29  *0.9 (5.4)* | 397.66  *4.6 (63.2)* | 973.44  *11.2 (65.7)* | 1676.41  *19.2 (28)* |
|  | *Total* | 15637.08 | 3306.78  *21.0 (99.1)* | 434.22  *2.8 (2.6)* | 635.97  *4.1 (67.9)* | 5541.04  *35.4 (69.4)* | 1407.49  *9.0 (97.8)* | 321.86  *2.1 (98.9)* | 1460.26  *9.3 (98.8)* | 2394.09  *15.3 (81.5)* | 117.25  *0.7 (29.9)* | 18.13  *0.7 (3.9)* |  | 29.65  *0.2 (3.4)* | 191.69  *1.2 (48.9)* | 67.61  *0.4 (5.7)* | 76.63  *0.5 (5.5)* | 614.22  *3.9 (97.7)* | 1427.39  *9.1 (96.3)* | 2407.19  *15.4 (40.3)* |
| 2050  RCP 2.6 | High | 1291.78 | 149.09  *11.5 (4.5)* | 14.46  *1.1 (0.1)* | 17.73  *1.4 (1.9)* | 137.82  *10.7 (1.7)* | 311.42  *24.1 (21.6)* | 64.40  *5.0 (19.8)* | 190.56  *14.8 (12.9)* | 405.87  *31.4 (13.8)* | 0.29  *0 (0.1)* | 0.12  *0 (0)* |  | 0.00  *0 (0)* | 3.36  *0.3 (0.9)* | 2.27  *0.2 (0.2)* | 0.02  *0 (0)* | 55.53  *4.3 (8.8)* | 98.50  *7.6 (6.6)* | 159.68  *12.4 (2.7)* |
|  | Moderate | 5138.23 | 1035.73  *20.2 (31)* | 130.03  *2.5 (0.8)* | 127.05  *2.5 (13.6)* | 1045.12  *20.3 (13.1)* | 681.72  *13.3 (47.4)* | 140.36  *2.7 (43.1)* | 843.89  *16.4 (57.1)* | 1115.24  *21.7 (38.0)* | 13.96  *0.3 (3.6)* | 5.14  *0.3 (1.1)* |  | 0.06  *0 (0)* | 42.08  *0.8 (10.7)* | 20.27  *0.4 (1.7)* | 1.55  *0 (0.1)* | 102.02  *2.0 (16.2)* | 316.01  *6.2 (21.3)* | 481.99  *9.4 (8.1)* |
|  | Low | 8090.52 | 1946.76  *24.1 (58.3)* | 387.06  *4.8 (2.4)* | 403.16  *5.0 (43.1)* | 3697.62  *45.7 (46.3)* | 242.11  *3.0 (16.8)* | 107.63  *1.3 (33.1)* | 419.15  *5.2 (28.3)* | 768.83  *9.5 (26.2)* | 105.36  *1.3 (26.8)* | 12.84  *1.3 (2.8)* |  | 80.21  *1.0 (9.1)* | 198.38  *2.5 (50.6)* | 112.81  *1.4 (9.5)* | 65.95  *0.8 (4.7)* | 277.78  *3.4 (44.2)* | 844.03  *10.4 (56.9)* | 1579.16  *19.5 (26.4)* |
|  | *Total* | 14520.53 | 3131.59  *21.6 (93.8)* | 531.55  *3.7 (3.2)* | 547.94  *3.8 (58.5)* | 4880.56  *33.6 (61.1)* | 1235.26  *8.5 (85.8)* | 312.39  *2.2 (96.0)* | 1453.60  *10.0 (98.3)* | 2289.94  *15.8 (78.0)* | 119.61  *0.8 (30.5)* | 18.10  *0.8 (3.9)* |  | 80.27  *0.6 (9.1)* | 243.83  *1.7 (62.2)* | 135.35  *0.9 (11.4)* | 67.53  *0.5 (4.8)* | 435.32  *3.0 (69.2)* | 1258.53  *8.7 (84.9)* | 2220.83  *15.3 (37.2)* |
| 2050  RCP 4.5 | High | 1182.01 | 181.37  *15.3 (5.4)* | 4.51  *0.4 (0)* | 10.68  *0.9 (1.1)* | 61.98  *5.2 (0.8)* | 554.97  *47.0 (38.6)* | 32.24  *2.7 (9.9)* | 81.08  *6.9 (5.5)* | 254.97  *21.6 (8.7)* | 0.14  *0 (0)* | 0.07  *0 (0)* |  | 0.00  *0 (0)* | 10.54  *0.9 (2.7)* | 3.53  *0.3 (0.3)* | 0.05  *0 (0)* | 27.00  *2.3 (4.3)* | 151.21  *12.8 (10.2)* | 192.33  *16.3 (3.2)* |
|  | Moderate | 4841.07 | 1265.33  *26.1 (37.9)* | 103.67  *2.1 (0.6)* | 176.25  *3.6 (18.8)* | 770.57  *15.9 (9.6)* | 535.87  *11.1 (37.2)* | 210.05  *4.3 (64.5)* | 656.63  *13.6 (44.4)* | 1108.45  *22.9 (37.8)* | 9.12  *0.2 (2.3)* | 5.13  *0.2 (1.1)* |  | 0.00  *0 (0)* | 47.08  *1.0 (12.0)* | 20.11  *0.4 (1.7)* | 2.06  *0 (0.1)* | 142.46  *2.9 (22.7)* | 321.16  *6.6 (21.7)* | 532.88  *11.0 (8.9)* |
|  | Low | 7886.08 | 1705.35  *21.6 (51.1)* | 381.83  *4.8 (2.3)* | 342.72  *4.3 (36.6)* | 3508.02  *44.5 (43.9)* | 211.32  *2.7 (14.7)* | 75.93  *1.0 (23.3)* | 704.65  *8.9 (47.7)* | 869.18  *11.0 (29.6)* | 77.80  *1.0 (19.8)* | 9.29  *1.0 (2.0)* |  | 2.57  *0 (0.3)* | 191.63  *2.4 (48.9)* | 110.81  *1.4 (9.3)* | 72.54  *0.9 (5.2)* | 254.09  *3.2 (40.4)* | 809.09  *10.3 (54.6)* | 1440.72  *18.3 (24.1)* |
|  | *Total* | 13909.16 | 3152.04  *22.7 (94.4)* | 490.01  *3.5 (3.0)* | 529.65  *3.8 (56.6)* | 4340.57  *31.2 (54.3)* | 1302.16  *9.4 (90.5)* | 318.21  *2.3 (97.8)* | 1442.36  *10.4 (97.5)* | 2232.60  *16.1 (76.0)* | 87.06  *0.6 (22.2)* | 14.49  *0.6 (3.1)* |  | 2.57  *0 (0.3)* | 249.25  *1.8 (63.6)* | 134.45  *1.0 (11.3)* | 74.65  *0.5 (5.3)* | 423.55  *3.0 (67.4)* | 1281.45  *9.2 (86.4)* | 2165.93  *15.6 (36.2)* |
| 2050  RCP 6.0 | High | 1110.70 | 124.43  *11.2 (3.7)* | 13.59  *1.2 (0.1)* | 16.00  *1.4 (1.7)* | 121.81  *11.0 (1.5)* | 347.94  *31.3 (24.2)* | 46.60  *4.2 (14.3)* | 106.82  *9.6 (7.2)* | 333.13  *30.0 (11.3)* | 0.34  *0 (0.1)* | 0.04  *0 (0)* |  | 0.00  *0 (0)* | 3.72  *0.3 (0.9)* | 1.65  *0.1 (0.1)* | 0.04  *0 (0)* | 26.37  *2.4 (4.2)* | 97.62  *8.8 (6.6)* | 129.40  *11.6 (2.2)* |
|  | Moderate | 5636.84 | 1310.00  *23.2 (39.2)* | 142.13  *2.5 (0.9)* | 216.70  *3.8 (23.1)* | 917.56  *16.3 (11.5)* | 749.95  *13.3 (52.1)* | 214.69  *3.8 (66.0)* | 872.94  *15.5 (59.0)* | 1189.81  *21.1 (40.5)* | 17.15  *0.3 (4.4)* | 5.91  *0.3 (1.3)* |  | 0.00  *0 (0)* | 38.50  *0.7 (9.8)* | 10.78  *0.2 (0.9)* | 2.57  *0 (0.2)* | 130.94  *2.3 (20.8)* | 413.17  *7.3 (27.9)* | 595.96  *10.6 (10.0)* |
|  | Low | 7978.52 | 1815.49  *22.8 (54.4)* | 312.98  *3.9 (1.9)* | 327.77  *4.1 (35.0)* | 3876.68  *48.6 (48.5)* | 276.98  *3.5 (19.2)* | 58.83  *0.7 (18.1)* | 476.41  *6.0 (32.2)* | 751.41  *9.4 (25.6)* | 73.74  *0.9 (18.8)* | 8.24  *0.9 (1.8)* |  | 0.74  *0 (0.1)* | 177.37  *2.2 (45.2)* | 89.06  *1.1 (7.5)* | 66.63  *0.8 (4.7)* | 324.08  *4.1 (51.5)* | 874.31  *11.0 (59.0)* | 1532.18  *19.2 (25.6)* |
|  | *Total* | 14726.06 | 3249.92  *22.1 (97.4)* | 468.71  *3.2 (2.8)* | 560.46  *3.8 (59.9)* | 4916.05  *33.4 (61.5)* | 1374.86  *9.3 (95.5)* | 320.11  *2.2 (98.4)* | 1456.18  *9.9 (98.5)* | 2274.34  *15.4 (77.5)* | 91.22  *0.6 (23.2)* | 14.20  *0.6 (3.1)* |  | 0.74  *0 (0.1)* | 219.59  *1.5 (56.0)* | 101.49  *0.7 (8.6)* | 69.23  *0.5 (4.9)* | 481.38  *3.3 (76.6)* | 1385.11  *9.4 (93.4)* | 2257.53  *15.3 (37.8)* |
| 2050  RCP 8.5 | High | 1380.81 | 159.67  *11.6 (4.8)* | 7.74  *0.6 (0)* | 7.58  *0.5 (0.8)* | 165.32  *12.0 (2.1)* | 533.46  *38.6 (37.1)* | 30.74  *2.2 (9.4)* | 153.55  *11.1 (10.4)* | 322.68  *23.4 (11.0)* | 0.07  *0 (0)* | 0.01  *0 (0)* |  | 0.00  *0 (0)* | 8.44  *0.6 (2.2)* | 1.29  *0.1 (0.1)* | 0.13  *0 (0)* | 41.61  *3.0 (6.6)* | 116.41  *8.4 (7.9)* | 167.89  *12.2 (2.8)* |
|  | Moderate | 5237.86 | 1113.67  *21.3 (33.4)* | 133.37  *2.5 (0.8)* | 136.22  *2.6 (14.5)* | 1118.97  *21.4 (14.0)* | 556.47  *10.6 (38.7)* | 172.42  *3.3 (53.0)* | 820.06  *15.7 (55.5)* | 1171.52  *22.4 (39.9)* | 11.43  *0.2 (2.9)* | 3.73  *0.2 (0.8)* |  | 0.00  *0 (0)* | 75.41  *1.4 (19.2)* | 27.28  *0.5 (2.3)* | 13.20  *0.3 (0.9)* | 164.12  *3.1 (26.1)* | 314.51  *6.0 (21.2)* | 594.52  *11.4 (9.9)* |
|  | Low | 8520.09 | 1877.54  *22 (56.3)* | 378.23  *4.4 (2.3)* | 324.96  *3.8 (34.7)* | 4182.29  *49.1 (52.4)* | 283.38  *3.3 (19.7)* | 107.65  *1.3 (33.1)* | 471.07  *5.5 (31.9)* | 826.02  *9.7 (28.1)* | 59.11  *0.7 (15.1)* | 9.85  *0.7 (2.1)* |  | 0.96  *0 (0.1)* | 171.63  *2 (43.8)* | 102.88  *1.2 (8.7)* | 129.89  *1.5 (9.3)* | 358.20  *4.2 (57.0)* | 913.16  *10.7 (61.6)* | 1676.71  *19.7 (28.1)* |
|  | *Total* | 15138.76 | 3150.87  *20.8 (94.4)* | 519.33  *3.4 (3.2)* | 468.76  *3.1 (50.1)* | 5466.58  *36.1 (68.4)* | 1373.31  *9.1 (95.4)* | 310.82  *2.1 (95.5)* | 1444.68  *9.5 (97.7)* | 2320.23  *15.3 (79)* | 70.60  *0.5 (18.0)* | 13.58  *0.5 (2.9)* |  | 0.96  *0 (0.1)* | 255.48  *1.7 (65.1)* | 131.45  *0.9 (11.1)* | 143.22  *0.9 (10.2)* | 563.93  *3.7 (89.7)* | 1344.08  *8.9 (90.7)* | 2439.12  *16.1 (40.8)* |
| 2070  RCP 2.6 | High | 1241.46 | 147.91  *11.9 (4.4)* | 15.18  *1.2 (0.1)* | 47.34  *3.8 (5.1)* | 106.99  *8.6 (1.3)* | 353.22  *28.5 (24.5)* | 88.96  *7.2 (27.3)* | 132.88  *10.7 (9.0)* | 346.67  *27.9 (11.8)* | 1.22  *0.1 (0.3)* | 1.10  *0.1 (0.2)* |  | 0.00  *0 (0)* | 4.92  *0.4 (1.3)* | 4.98  *0.4 (0.4)* | 0.06  *0 (0)* | 48.23  *3.9 (7.7)* | 106.22  *8.6 (7.2)* | 164.42  *13.2 (2.8)* |
|  | Moderate | 5108.22 | 1201.35  *23.5 (36.0)* | 125.93  *2.5 (0.8)* | 209.68  *4.1 (22.4)* | 975.91  *19.1 (12.2)* | 667.45  *13.1 (46.4)* | 178.93  *3.5 (55.0)* | 679.93  *13.3 (46.0)* | 1045.73  *20.5 (35.6)* | 14.79  *0.3 (3.8)* | 8.53  *0.3 (1.8)* |  | 0.00  *0 (0)* | 73.81  *1.4 (18.8)* | 21.67  *0.4 (1.8)* | 2.59  *0.1 (0.2)* | 120.49  *2.4 (19.2)* | 384.82  *7.5 (26)* | 603.38  *11.8 (10.1)* |
|  | Low | 8114.29 | 1805.11  *22.2 (54.1)* | 347.16  *4.3 (2.1)* | 256.77  *3.2 (27.4)* | 3844.72  *47.4 (48.1)* | 272.12  *3.4 (18.9)* | 51.17  *0.6 (15.7)* | 630.19  *7.8 (42.6)* | 829.25  *10.2 (28.2)* | 72.59  *0.9 (18.5)* | 5.22  *0.9 (1.1)* |  | 0.07  *0 (0)* | 176.12  *2.2 (44.9)* | 101.26  *1.2 (8.5)* | 112.22  *1.4 (8.0)* | 335.05  *4.1 (53.3)* | 809.14  *10.0 (54.6)* | 1533.86  *18.9 (25.7)* |
|  | *Total* | 14463.97 | 3154.37  *21.8 (94.5)* | 488.27  *3.4 (3)* | 513.78  *3.6 (54.9)* | 4927.62  *34.1 (61.7)* | 1292.78  *8.9 (89.8)* | 319.06  *2.2 (98)* | 1442.99  *10.0 (97.6)* | 2221.65  *15.4 (75.7)* | 88.60  *0.6 (22.6)* | 14.84  *0.6 (3.2)* |  | 0.07  *0 (0)* | 254.85  *1.8 (65.0)* | 127.90  *0.9 (10.8)* | 114.87  *0.8 (8.2)* | 503.77  *3.5 (80.1)* | 1300.19  *9.0 (87.7)* | 2301.66  *15.9 (38.5)* |
| 2070  RCP 4.5 | High | 1320.69 | 201.84  *15.3 (6.0)* | 5.85  *0.4 (0)* | 26.52  *2.0 (2.8)* | 100.94  *7.6 (1.3)* | 514.19  *38.9 (35.7)* | 36.33  *2.8 (11.2)* | 127.62  *9.7 (8.6)* | 303.74  *23.0 (10.3)* | 1.01  *0.1 (0.3)* | 2.66  *0.1 (0.6)* |  | 0.00  *0 (0)* | 9.42  *0.7 (2.4)* | 4.26  *0.3 (0.4)* | 0.02  *0 (0)* | 33.21  *2.5 (5.3)* | 131.72  *10.0 (8.9)* | 178.64  *13.5 (3.0)* |
|  | Moderate | 5007.23 | 1296.22  *25.9 (38.8)* | 99.67  *2.0 (0.6)* | 232.66  *4.6 (24.8)* | 738.74  *14.8 (9.2)* | 561.73  *11.2 (39.0)* | 189.16  *3.8 (58.1)* | 790.33  *15.8 (53.5)* | 1076.40  *21.5 (36.7)* | 14.86  *0.3 (3.8)* | 7.46  *0.3 (1.6)* |  | 0.04  *0 (0)* | 47.96  *1.0 (12.2)* | 19.60  *0.4 (1.7)* | 0.63  *0 (0)* | 143.38  *2.9 (22.8)* | 313.46  *6.3 (21.1)* | 525.06  *10.5 (8.8)* |
|  | Low | 7808.02 | 1722.72  *22.1 (51.6)* | 373.11  *4.8 (2.3)* | 294.19  *3.8 (31.4)* | 3628.05  *46.5 (45.4)* | 238.01  *3.0 (16.5)* | 93.70  *1.2 (28.8)* | 526.57  *6.7 (35.6)* | 856.37  *11.0 (29.2)* | 70.82  *0.9 (18.0)* | 4.46  *0.9 (1.0)* |  | 23.98  *0.3 (2.7)* | 156.14  *2.0 (39.8)* | 72.50  *0.9 (6.1)* | 45.34  *0.6 (3.2)* | 321.88  *4.1 (51.2)* | 855.67  *11.0 (57.7)* | 1475.51  *18.9 (24.7)* |
|  | *Total* | 14135.94 | 3220.79  *22.8 (96.5)* | 478.62  *3.4 (2.9)* | 553.37  *3.9 (59.1)* | 4467.73  *31.6 (55.9)* | 1313.94  *9.3 (91.3)* | 319.19  *2.3 (98.1)* | 1444.52  *10.2 (97.7)* | 2236.51  *15.8 (76.2)* | 86.69  *0.6 (22.1)* | 14.58  *0.6 (3.2)* |  | 24.02  *0.2 (2.7)* | 213.52  *1.5 (54.4)* | 96.36  *0.7 (8.1)* | 46.00  *0.3 (3.3)* | 498.47  *3.5 (79.3)* | 1300.85  *9.2 (87.8)* | 2179.21  *15.4 (36.5)* |
| 2070  RCP 6.0 | High | 1313.54 | 186.66  *14.2 (5.6)* | 6.29  *0.5 (0)* | 11.03  *0.8 (1.2)* | 131.37  *10.0 (1.6)* | 558.49  *42.5 (38.8)* | 21.66  *1.6 (6.7)* | 102.62  *7.8 (6.9)* | 291.67  *22.2 (9.9)* | 0.84  *0.1 (0.2)* | 2.91  *0.1 (0.6)* |  | 0.00  *0 (0)* | 14.16  *1.1 (3.6)* | 2.27  *0.2 (0.2)* | 0.01  *0 (0)* | 23.24  *1.8 (3.7)* | 146.72  *11.2 (9.9)* | 186.40  *14.2 (3.1)* |
|  | Moderate | 4479.66 | 1141.55  *25.5 (34.2)* | 105.67  *2.4 (0.6)* | 153.28  *3.4 (16.4)* | 829.28  *18.5 (10.4)* | 491.32  *11.0 (34.1)* | 161.47  *3.6 (49.6)* | 588.46  *13.1 (39.8)* | 992.74  *22.2 (33.8)* | 9.34  *0.2 (2.4)* | 6.55  *0.2 (1.4)* |  | 0.00  *0 (0)* | 53.81  *1.2 (13.7)* | 19.91  *0.4 (1.7)* | 2.16  *0 (0.2)* | 127.59  *2.8 (20.3)* | 284.10  *6.3 (19.2)* | 487.58  *10.9 (8.2)* |
|  | Low | 7584.25 | 1688.97  *22.3 (50.6)* | 403.72  *5.3 (2.5)* | 334.53  *4.4 (35.7)* | 3171.80  *41.8 (39.7)* | 193.87  *2.6 (13.5)* | 126.60  *1.7 (38.9)* | 721.06  *9.5 (48.8)* | 876.82  *11.6 (29.9)* | 62.63  *0.8 (16.0)* | 4.24  *0.8 (0.9)* |  | 31.30  *0.4 (3.5)* | 173.33  *2.3 (44.2)* | 92.34  *1.2 (7.8)* | 73.90  *1.0 (5.3)* | 293.65  *3.9 (46.7)* | 696.12  *9.2 (47.0)* | 1360.64  *17.9 (22.8)* |
|  | *Total* | 13377.45 | 3017.18  *22.6 (90.4)* | 515.68  *3.9 (3.1)* | 498.85  *3.7 (53.3)* | 4132.46  *30.9 (51.7)* | 1243.68  *9.3 (86.4)* | 309.73  *2.3 (95.2)* | 1412.13  *10.6 (95.5)* | 2161.23  *16.2 (73.6)* | 72.81  *0.5 (18.5)* | 13.70  *0.5 (3.0)* |  | 31.30  *0.2 (3.5)* | 241.29  *1.8 (61.5)* | 114.52  *0.9 (9.7)* | 76.07  *0.6 (5.4)* | 444.49  *3.3 (70.7)* | 1126.94  *8.4 (76.0)* | 2034.62  *15.2 (34.0)* |
| 2070  RCP 8.5 | High | 1228.92 | 166.66  *13.6 (5.0)* | 12.01  *1.0 (0.1)* | 31.82  *2.6 (3.4)* | 94.16  *7.7 (1.2)* | 438.54  *35.7 (30.5)* | 54.18  *4.4 (16.6)* | 164.03  *13.3 (11.1)* | 265.86  *21.6 (9.1)* | 0.70  *0.1 (0.2)* | 0.96  *0.1 (0.2)* |  | 0.00  *0 (0)* | 1.36  *0.1 (0.3)* | 2.00  *0.2 (0.2)* | 0.24  *0 (0)* | 33.81  *2.8 (5.4)* | 108.79  *8.9 (7.3)* | 146.19  *11.9 (2.4)* |
|  | Moderate | 5648.26 | 1292.19  *22.9 (38.7)* | 141.28  *2.5 (0.9)* | 238.49  *4.2 (25.5)* | 1015.76  *18.0 (12.7)* | 651.79  *11.5 (45.3)* | 206.30  *3.7 (63.4)* | 879.71  *15.6 (59.5)* | 1199.18  *21.2 (40.8)* | 16.30  *0.3 (4.2)* | 7.25  *0.3 (1.6)* |  | 0.00  *0 (0)* | 54.77  *1.0 (14.0)* | 16.21  *0.3 (1.4)* | 15.84  *0.3 (1.1)* | 213.32  *3.8 (33.9)* | 343.06  *6.1 (23.1)* | 643.20  *11.4 (10.8)* |
|  | Low | 8435.83 | 1788.28  *21.2 (53.6)* | 334.71  *4.0 (2.0)* | 391.69  *4.6 (41.8)* | 4091.83  *48.5 (51.2)* | 278.72  *3.3 (19.4)* | 58.96  *0.7 (18.1)* | 414.80  *4.9 (28.1)* | 915.28  *10.8 (31.2)* | 151.22  *1.8 (38.5)* | 10.33  *1.8 (2.2)* |  | 4.17  *0 (0.5)* | 189.56  *2.2 (48.3)* | 99.43  *1.2 (8.4)* | 99.24  *1.2 (7.1)* | 372.00  *4.4 (59.2)* | 869.36  *10.3 (58.6)* | 1633.76  *19.4 (27.3)* |
|  | *Total* | 15313.01 | 3247.13  *21.2 (97.3)* | 488.00  *3.2 (3.0)* | 662.00  *4.3 (70.7)* | 5201.75  *34.0 (65.1)* | 1369.05  *8.9 (95.1)* | 319.45  *2.1 (98.2)* | 1458.54  *9.5 (98.6)* | 2380.32  *15.5 (81.1)* | 168.23  *1.1 (42.9)* | 18.54  *1.1 (4.0)* |  | 4.17  *0 (0.5)* | 245.68  *1.6 (62.6)* | 117.63  *0.8 (9.9)* | 115.33  *0.8 (8.2)* | 619.13  *4.0 (98.5)* | 1321.21  *8.6 (89.1)* | 2423.15  *15.8 (40.5)* |
| IUCN | Extant | 6018.68 | 1594.54  *26.5 (47.8)* | 1179.97  *19.6 (7.2)* | 3.09  *0.1 (0.3)* | 1256.61  *20.9 (15.7)* | 623.52  *10.4 (43.3)* | 41.93  *0.7 (12.9)* | 737.57  *12.3 (49.9)* | 572.64  *9.5 (19.5)* | 3.76  *0.1 (1.0)* | 5.06  *0.1 (1.1)* |  | 0.00  *0 (0)* | 0.00  *0 (0)* | 0.00  *0 (0)* | 0.00  *0 (0)* | 7.86  *0.1 (1.2)* | 732.63  *12.2 (49.4)* | 740.49  *12.3 (12.4)* |

^1^ The area in km2 in normal font and its meaning in percentage (%) in italics. Without parentheses the percentage (%) with respect to the area of the potential habitat range and in parentheses the percentage (%) with respect to the area of the NPA modalities

^2^ B-aY: Yunga Altimontane (Pluvial) Forest, B-bY: Yunga Basimontane Forest, Bes-in: Seasonally dry inter-Andean forest, B-mY: Yunga mountain forest, Jal: Jalca, Ma: Andean scrub, PH: Grasslands/Herbazales, Vsec: Secondary Vegetation, Agri: Agricultural Zone, and others.

^3^ NP: National Park, NS: National Sanctuary, CR: Communal Reserves, RZ: Reserved Areas, RCA: Regional Conservation Areas, PCA: Private Conservation Areas.


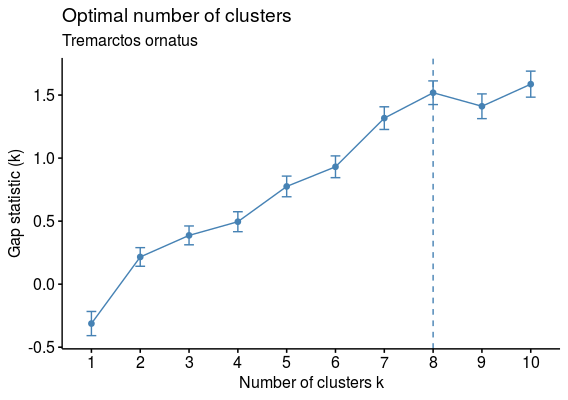


(a)


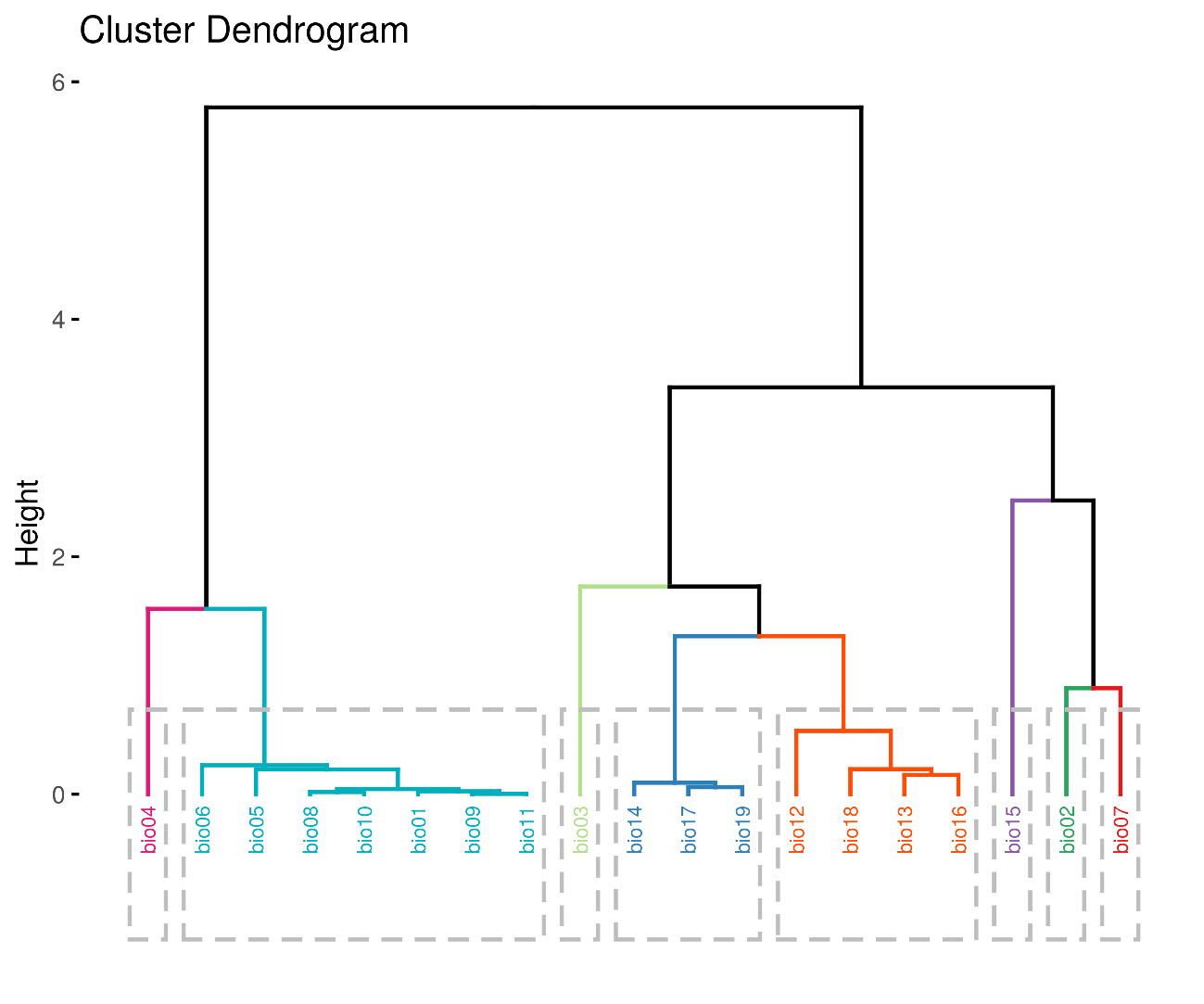


(b)

**Figure S1. (a)** Optimal number of clusters and **(b)** Cluster dendrogram between the bioclimatic variables for the modeling of the potential distribution of *T. ornatus a* in Amazonas (Peru).


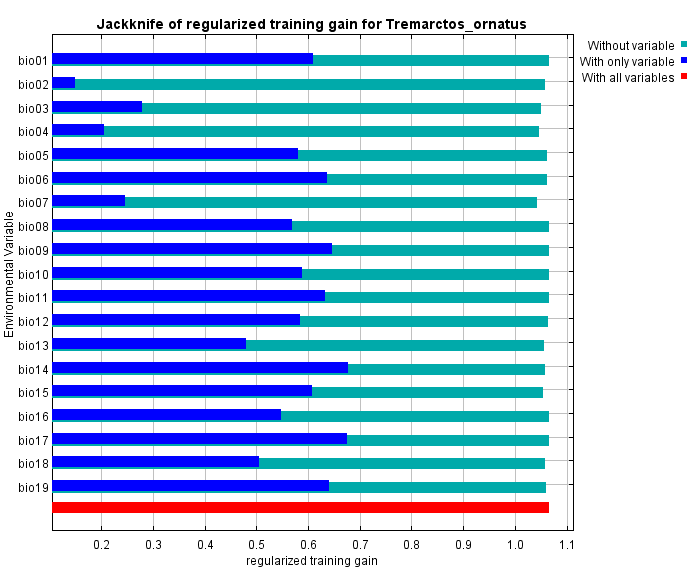


**Figure S3.** Jackknife test in a preliminary model generated using only the 19 bioclimatic variables for the modeling of the potential distribution of *T. ornatus a* in Amazonas (Peru). Regularized training gain without variable (green), with only variable (blue) and with all variables (red).
